# Vowel dyslexia in Turkish: A window to the complex structure of the sublexical route

**DOI:** 10.1371/journal.pone.0249016

**Published:** 2021-03-24

**Authors:** Selçuk Güven, Naama Friedmann

**Affiliations:** 1 Department of Speech and Language Therapy, Anadolu University, Eskişehir, Turkey; 2 Language and Brain Lab, Tel Aviv University, Tel Aviv, Israel; University of Trento, ITALY

## Abstract

We report on developmental vowel dyslexia, a type of dyslexia that selectively affects the reading aloud of vowel letters. We identified this dyslexia in 55 Turkish-readers aged 9–10, and made an in-depth multiple-case analysis of the reading of 17 participants whose vowel dyslexia was relatively selective. These participants made significantly more vowel errors (vowel substitution, omission, migration, and addition) than age-matched controls, and significantly more errors in vowel letters than in consonants. Vowel harmony, a pivotal property of Turkish phonology, was intact and the majority of their vowel errors yielded harmonic responses. The transparent character of Turkish orthography indicates that vowel dyslexia is not related to ambiguity in vowel conversion. The dyslexia did not result from a deficit in the phonological-output stage, as the participants did not make vowel errors in nonword repetition or in repeating words they had read with a vowel error. The locus of the deficit was not in the orthographic-visual-analyzer either, as their same-different decision on words differing in vowels was intact, and so was their written-word comprehension. They made significantly more errors on nonwords than on words, indicating that their deficit was in vowel processing in the sublexical route. Given that their single-vowels conversion was intact, and that they showed an effect of the number of vowels, we conclude that their deficit is in a vowel-specific buffer in the sublexical route. They did not make vowel errors within suffixes, indicating that suffixes are converted as wholes in a separate sublexical sub-route. These results have theoretical implications for the dual-route model: they indicate that the sublexical route converts vowels and consonants separately, that the sublexical route includes a vowel buffer, and a separate morphological conversion route. The results also indicate that types of dyslexia can be detected in transparent languages given detailed error-analysis and dyslexia-relevant stimuli.

## 1. Introduction

Cognitive models and selective language impairments go hand in hand through the history of cognitive neuropsychology. Cognitive models allow the understanding of the nature of specific impairments, and predicting patterns of deficits. Selective impairments, from their side, contribute a way to examine cognitive models, provide constraints, decide between competing models, and force fine-tuning and changes in models. The study of dyslexias is one example for exactly such dynamic: the description of three types of dyslexia by Marshall and Newcombe [1] came hand in hand with a suggestion of a dual-route model for word reading (building partially on Morton’s [2] model for lexical retrieval, which in itself was also built on detailed descriptions of lexical retrieval impairments). This model then became more and more detailed and polished in light of new discoveries in the dyslexia domain [3–14].

**Fig 1:**
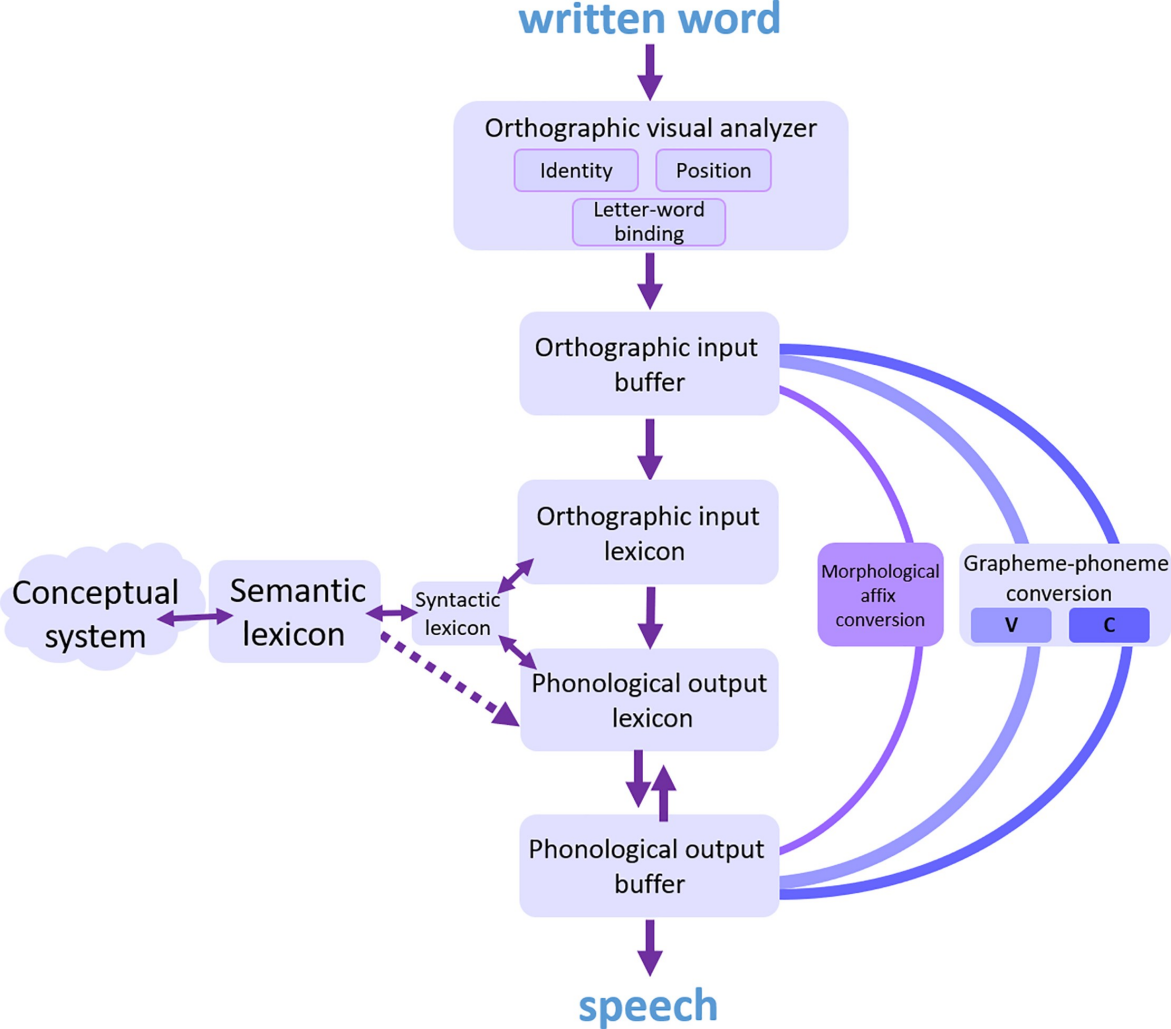
A dual-route reading model.

The view that emerged in the past 50 years, which created a model that can explain all kinds of dyslexia we currently know (a current version is brought in Fig 1), is that of a process that starts in an orthographic-visual analysis stage. This stage is responsible for letter identification, letter position encoding, and letter-to-word binding [7]. The information then flows to an orthographic input buffer, which holds this information for a short time and parses the input string into graphemes; this is probably also where the morphological analysis is performed [15]. This information then flows in two routes: One is a lexical route, which includes an orthographic input lexicon and a phonological output lexicon, which hold orthographic and phonological representations of words that the reader already knows, respectively. The phonological representation then arrives in a phonological output buffer, a short-term component that holds all the phonological information until production, and assembles the phonological units (phonemes, morphemes, functions words). The lexical route also includes a branch connecting the orthographic input lexicon to the semantic lexicon, which allows for the comprehension of written words. The other route is a sublexical route, which converts graphemes into phonemes according to the grapheme-to-phoneme rules of the language [16]. The converted phonemes from the sublexical route arrive in the phonological output buffer, where they are held and assembled. Nonwords can only be read via the sublexical route, as they have no representation in the lexicons, whereas words can be read via both routes, but the accurate and faster route is the lexical one.

Data coming from dyslexia research in recent years challenged the view of the sublexical route as a component that linearly converts whole graphemes into phonemes. Dyslexias that affect only the conversion of one phonological feature, such as voicing [17] indicated that consonant letters may be converted to bundles of phonological features rather than whole phonemes. Data showing that morphological affixes are treated as pre-assembled whole units in the phonological output buffer [18] and in the orthographic input buffer [19] suggested that the sublexical route may treat morphological affixes separately, and convert them, at least in Hebrew, from a whole written affix to a whole phonological affix [15] (see Fig 1).

Finally, vowel letter dyslexia, a dyslexia stemming from a sublexical route impairment that selectively affects vowels, sheds further light on the sublexical route. Khentov-Kraus and Friedmann [20] reported on 23 Hebrew readers (one with acquired, 22 with developmental dyslexia) who, when reading via the sublexical route (when they read nonwords or when they were forced to read words sublexically because of a lexical route deficit) made errors (migration, omission, addition, or substitution) in vowel letters, but not in consonants. A similar pattern was also reported for Arabic [9]. These findings suggested that the sublexical route processes vowels and consonants separately. Khentov-Kraus and Friedmann identified the locus of impairment in a vowel buffer in the sublexical route.

These insights came from Hebrew and Arabic, orthographies in which vowel letters are not consistently represented in the orthography, and in which vowel letters are polyphonic- they can be converted to one of several phonemes. It would be interesting to find out whether vowel dyslexia can also be identified in an orthographically transparent language, such as Turkish. Finding vowel dyslexia in Turkish, in which vowel letters are converted transparently and unambiguously to vowel phonemes, will indicate that it is not the specific properties of vowels in Semitic orthographies that gave rise to vowel dyslexia, and would support a change in the description of the sublexical route, according to which vowel letters and consonant letters are processed separately in it.

### 1.1. A brief description of vowels in Turkish

The modern orthography of Turkish is composed of a 29-letter alphabet consisting of eight vowels and 21 consonants, based on a modified Latin script. In most cases, a single phoneme is represented with a single letter, and the grapheme-to-phoneme correspondence is highly consistent and transparent. The only exceptions are words borrowed from other languages, which are usually transferred into Turkish with their original phonology. For example, the word “katip”, which is borrowed from Arabic, is written with a single *a* but read, like in the Arabic origin, with a long *a*, /kaatip/; vowel geminates are very rare in the Turkish orthography (consonant geminates do exist). Turkish syllable structure is mainly canonical (CV, VC, CVC, or VCV), but still allows complex word-initial and word-final consonant clusters. Turkish has a regular final stress position (with some exceptional loanwords). There are eight vowels in Turkish, characterized by the features front and back, high and low, and rounded and unrounded, as summarized in Table 1.

**Table 1:** Features of Turkish vowels and vowel harmony.

	Front		Back	
	Unrounded	Rounded	Unrounded	Rounded
High	i	ü	ı	u
Low	e	ö	a	o

Turkish has a specific phonological property called **vowel harmony**. Vowels in the same word tend to belong to the same vowel class, defined by fronting and rounding. Front vowels must be followed by front vowels, and back vowels must be followed by back vowels; and if a vowel is a high vowel, the vowel that follows it must be rounded. Therefore, Turkish words typically include either vowels from the group {i, e, ü, ö}, or vowels from the group {ı, a, u, o}. These considerations apply not only stem-internally, but also for morphological suffixes: when a morphological suffix is added to a stem, it conforms to the vowels of the stem, e.g., the plural suffix "lar" has two allomorphs: *lar* and *ler*. When added to a stem like "kadın"(woman) it becomes "kadınlar"(women), but when added to a word like "çilek"(strawberry) with the other vowel set, it becomes "çilekler"(strawberries). Because Turkish is morphologically very rich, and all morphological affixes have allomorphs and are subject to vowel harmony, the phenomenon of vowel harmony is very central in Turkish phonology and morpho-phonology. Most of the exceptions to these rules, disharmonic words, occur in loanwords.

Studies of the acquisition of vowel harmony in Turkish found that vowel harmony is acquired early [21]. Aksu-Koç and Slobin [22] show that around the age of 2;0 Turkish-speaking children use this rule accurately.

The role of vowel harmony in reading processes has not been studied widely. Raman and Weekes [23], who reported BRB, a Turkish speaker with acquired dysgraphia, reported that although BRB had a considerable phonological deficit in reading and writing, he rarely violated vowel harmony.

### 1.2. Dyslexia in Turkish

There are very few studies on types of developmental dyslexia in Turkish. In a recent study, Güven and Friedmann [24] investigated letter position dyslexia in Turkish, a specific type of dyslexia that results from a deficit in letter position encoding, causing letter transpositions within words [9, 25–30]. One other study described acquired dyslexia in Turkish which apparently affected the phonological output stages [31]. Other types of dyslexia–developmental or acquired–have not been reported for Turkish yet. The few papers that examined developmental dyslexia in Turkish (without distinguishing between different dyslexia types), worked under the assumption that dyslexia in Turkish mainly affects reading fluency [32–34], and have not characterized the types of errors individuals with dyslexia make. A similar approach was advocated also regarding other transparent orthographies in which dyslexia was said not to occur at all [35] or to only manifest itself in fluency measures [36–38]. Studies of reading development in typical Turkish readers focus mainly on the contribution of phonological abilities to reading and spelling acquisition [39, 40].

The current study, therefore, aims to start filling the gap by reporting and exploring in detail vowel dyslexia in Turkish. This would allow us to examine the conclusion regarding the suggested modification in the sublexical route, according to which vowels are processed separately from consonants, but this time from the perspective of a transparent orthography with a consistent representation of vowels. It will also allow us to examine a common perception according to which there are no dyslexias in transparent languages like Turkish, or that dyslexia in Turkish (and other transparent languages) can only be detected through measures of fluency, not by error analysis [32, 34, 36]. The special characteristics of Turkish, including vowel harmony, transparency, and rich morphology would allow us to ask questions about the properties of vowel dyslexia that have not been tested so far and to examine further the structure of the sublexical route.

## 2. Identifying individuals with developmental vowel dyslexia

### 2.1. Participants

The participants with vowel dyslexia in this study were identified through a school-wide reading testing in which we administered tests of reading aloud from the FRİGÜ reading battery [41] to 320 Turkish-reading children aged 9–10 in six schools, as well as to approximately 40 children referred to us by their teachers who suspected they had learning or reading difficulties.

#### 2.1.1 Control groups

Of the 320 children in the school-wide assessment, which included both children with typical reading and children with dyslexia, we had initially selected for the control group for the screening test 240 children for whom the teacher did not report any reading or learning difficulty. After the administration of the screening task to these 240 children, we excluded 35 from the control group because they were outliers, according to their total number of errors in the screening task, which was 3SD from the group average (so they apparently had a deficit that the teachers were not aware of). This procedure yielded a control group for the screening test of 205 4^th^ graders, 111 girls and 94 boys, with no report of reading disability.

The control group for the further vowel dyslexia tests described below was a group of 60 children taken from this control group of 205 children (the children in the school-wide assessment who had no report of difficulties, and who were not outliers). These 60 children were 28 females and 32 males, fourth-graders aged 9 to 10 years, who according to their teacher had no speech, language, hearing, or cognitive problems, and who had typical language according to the clinical opinion of the speech-language pathologists testing them.

### 2.2. Procedure

Each of the participants was tested individually in a quiet room: The participants in the school-wide assessment were tested in a quiet room in their school; the children who were referred to our clinic were tested in the clinic’s testing rooms. In the screening test as well as in all the vowel dyslexia tests reported below, all stimuli were displayed as lists presented on a white page in 14 pt. font, with double vertical spacing between words. No time limit was imposed during testing, the written lists remained in front of the participants for as long as they needed, and no response-contingent feedback was given by the experimenter.

All methods of the study were performed in accordance with the Declaration of Helsinki. Ethical approval for this study was granted by the Anadolu University Research Ethics Committee.

### 2.3. The screening test used to identify children with vowel dyslexia

For the initial identification of individuals with vowel dyslexia, and for the exclusion of individuals with other types of dyslexia, we first administered the screening test from the FRİGÜ test battery, which was developed to identify types of dyslexia in Turkish. The screening part of the FRİGÜ is an oral reading test that includes three blocks: 151 single words, 60 word pairs, and 40 nonwords.

The word and nonword lists of the FRİGÜ screening test were constructed so that they include words that are sensitive to various types of dyslexia; words with different stress patterns or with an ambiguous grapheme-to-phoneme conversion for identifying surface dyslexia; function words and morphologically complex words to identify phonological output buffer dyslexia, orthographic input buffer dyslexia, and deep dyslexia; abstract words for deep dyslexia; words (and nonwords) with many orthographic neighbors for identifying visual dyslexia and letter identity dyslexia; words (and nonwords) that can be read as other words by neglecting one side of the word, for identifying neglect dyslexia; and migratable words for the identification of letter position dyslexia. The nonwords were included for identifying phonological and deep dyslexia as well as various peripheral dyslexias; the word pairs were constructed such that between-word migrations create other existing words, to enable the detection of attentional dyslexia. Importantly, the screening test also included words and nonwords in which vowel errors (vowel letter migration, substitution, omission, or addition) create other words and hence could be sensitive to vowel letter dyslexia, 100 such words and 29 nonwords. (See S1 Table for an overview of all tests from the FRİGÜ test battery used in the current study, their properties, and the number of control participants who read them).

In addition to the screening test, which we used for the total number of errors and for excluding other types of dyslexia, we also ran a targeted vowel dyslexia test from the FRİGÜ battery, the ÜZÜM test ([41], reported in detail in Section 3 below), which we used for the analysis of the rate of errors in vowel letters in comparison to the control group.

### 2.4. Results: 55 children with developmental vowel dyslexia

The analysis of the screening test for a total number of errors, and of the ÜZÜM vowel dyslexia test for the rate of errors in vowel letters, indicated that 55 children had vowel dyslexia. They had dyslexia according to the screening test, as they made significantly more errors in total in the words and nonwords lists of the screening test compared to the control group (using 1-tailed Crawford & Howell’s t-test [42]), and, according to the ÜZÜM vowel dyslexia nonword test, they made significantly more vowel letter errors compared to their age-matched control group. (The rates of vowel errors of these 55 participants with vowel dyslexia in the ÜZÜM vowel dyslexia word and nonword tests are reported in S1 Fig).

The wider context of our research plan included 155 children with dyslexia, so the finding that 55 of these dyslexic children had vowel dyslexia (sometimes in addition to other dyslexia types) suggests that vowel dyslexia is quite a common type of dyslexia in Turkish.

## 3. In-depth exploration of vowel dyslexia: 17 participants

### 3.1. General methods

#### 3.1.1. The 17 participants with vowel dyslexia who participated in further testing

Beyond demonstrating that vowel dyslexia can be identified in Turkish, we were mainly interested in exploring the properties of vowel dyslexia in Turkish. For this aim, we wanted to focus on a vowel dyslexia-specific group, and refrain from the effects of other impairments. Therefore, we excluded from the 55 participants with vowel dyslexia the ones whose dyslexia was less specific–those who made more consonant errors than vowel errors in one of the tests, or those who showed more errors that are characteristic of other types of dyslexia–and remained with 17 children who had relatively selective vowel dyslexia.

We included participants in the specific-vowel-dyslexia group for the in-depth analysis of the properties and functional locus of vowel dyslexia if they met the following inclusion criteria:

1. Made significantly more reading errors in total in the FRİGÜ screening test compared with the control group.
2. Made significantly more vowel letter errors (omission, addition, substitution, and migration of vowel letters) than the control group in reading nonwords in the ÜZÜM vowel dyslexia test (described in detail below in Section 3.3).
3. Made significantly more vowel letter errors than consonant errors in the ÜZÜM words and nonwords tests.
4. Made more vowel letter errors than any other type of error (letter identity, letter position, migration between words, morphological errors) in the screening test. (One participant, SS, also had letter position dyslexia but her vowel letter errors, when excluding her letter transpositions, which could arise from letter position dyslexia, were still significantly above the controls so we included her). We did not exclude individuals who had surface dyslexia in addition to vowel dyslexia, see Section 3.4.3).
5. Agreed to participate in further testing sessions.

These criteria yielded 17 participants with a relatively selective vowel dyslexia who participated in further testing. These 17 participants were all monolingual Turkish-speaking children in 4^th^ grade, aged 9–10, 6 males and 11 females. All of them were right-handed. All of the participants were living in Eskişehir, Turkey, and were pupils in regular schools and regular classes. According to the reports of their parents and/or teachers, and according to the informal observation made by speech-language pathologists who were administering the reading tests, none of them had speech and language disorders beyond their reading difficulties, nor any history of brain lesions, neurological condition, or cognitive problems. None of them had been previously officially diagnosed with dyslexia or learning disability, but when we discussed their reading with their teachers, the teachers of almost all of them reported they had difficulties and expressed concerns about the reading.

Of the 17 children with vowel dyslexia, 15 children were selected from the school-wide reading testing, and the other two children were recruited from teachers who referred them to us because they suspected they had learning or reading difficulties.

#### 3.1.2. Procedure

Each of the participants took part in at least 8 tests, which were administered in several sessions. The number of sessions and length of each session were determined by each of the participants. Informed consent was provided from all participants’ parents or legal guardians prior to undertaking the testing procedures.

We administered the ÜZÜM vowel dyslexia test battery, which we developed to examine the nature of this dyslexia, and the way it manifests itself in Turkish. We report below the results of the ÜZÜM test of reading aloud of a word list, followed by a nonword list, and an in-depth analysis of vowel letter errors and their characteristics. With the aim of identifying the locus in the reading process that gives rise to our participants’ vowel dyslexia, we then report on results of silent reading tests from the ÜZÜM vowel dyslexia battery, single letter conversion, and tests of phonological output that do not involve reading.

#### 3.1.3. Error coding and analysis

The analysis of reading errors was guided by the following principles:

1. Errors of vowel letter omission, substitution, and addition were counted as vowel letter errors.
2. Errors of consonant letter omission, substitution, and addition were counted as consonant letter errors.
3. Transpositions of two consonants were counted as a consonant error and transpositions of two vowels or a vowel and a consonant were counted as a vowel error.
4. In cases where the child produced a sequence of responses to a target stimuli, and one of these responses was an error, we counted the item as incorrect and analyzed the first erroneous response.

#### 3.1.4. Statistical analyses

For all the analyses in which we examined whether each participant with dyslexia made significantly more errors than their age-matched control group, we used one-tailed Crawford and Howell’s [42] t-test (we used this test for the comparison for each of the error types we examined). Within-participant comparisons between two conditions were conducted using chi-square tests (2-tailed comparisons). At the group level, comparisons between two conditions were conducted using the Wilcoxon signed-rank test. Comparisons at the group level between the vowel dyslexia group and the large control group were done using Welch’s *t*-test. Correlations were assessed using Pearson correlation coefficient. Effect sizes for Welch’s t-test and Wilcoxon test are reported with *Cohen’s d and Hedges’ g*. An alpha level of 0.05 was used in all comparisons. For the analyses of the effects of word familiarity, we followed the recommendation of Gries [43, 44] and used dispersion instead of frequency. The database we used [45] calculated dispersion values as Julliand D dispersion index, which takes a value ranging from 0 to 1, where 1 is the word most evenly distributed across the corpus, and 0.01 indicated that the word only occurs in an extremely small part of the corpus. In all but 5 sections, we report error percentages out of the total number of words; in the 5 sections in which we refer to a different denominator (e.g., out of the total number of errors), the denominator is stated.

### 3.2. Oral reading of words

#### 3.2.1. Experimental stimuli: Word reading

The üzüm word list included 124 words, 4-to-7 letters long (*M* = 4.9, *SD* = 0.9). These words were selected so that in each of them, at least one vowel letter error creates an existing word (see Table 2 for the various types of vowel errors, exemplified with errors that the participants with vowel dyslexia made in the ÜZÜM and screening tests). For the comparison of vowel and consonant errors, 99 of the words had lexical potentials both for vowel- and for consonant errors, namely, in these words at least one vowel letter error results in an existing word, and at least one consonant letter error results in an existing word.

**Table 2:** Examples for vowel errors of various types that the vowel dyslexia participants made in the üzüm word and nonword tests.

Condition	Target word	Response with vowel error	Translation target word	Translation response	Example from English
***Target words***					
***Vowel substitution***	kara	kare	black	square	form-farm
***Vowel omission***	ada	ad	island	name	forum-form
***Vowel migration***	sade	seda	simple	voice	bate-beta
***Vowel addition***	çil	çile	speckle	suffering	form-forum
***Vowel addition (doubling)***	damdaki	damadaki	the one on the roof	the one in the checkers game	tent-tenet
***Target nonwords***					
***Vowel substitution***	yıra	yara		scar	
***Vowel omission***	kazo	kaz		goose	
***Vowel migration***	kerük	kürek		shovel	
***Vowel addition (doubling)***	kilem	ikilem			

For the analyses of the effect of vowel harmony on vowel errors, the list included 20 nonharmonic words in which one vowel error can create an existing harmonic word, and another vowel error can create an existing nonharmonic word.

To be able to compare between errors in vowels that are part of the stem and errors in vowels that function as a suffix (or part thereof), the list included 120 words in which an error in a vowel letter of the stem creates another existing word and 55 words in which an error in a vowel letter in the suffix could create another existing word.

#### 3.2.2. Results: Word reading

**Fig 2:**
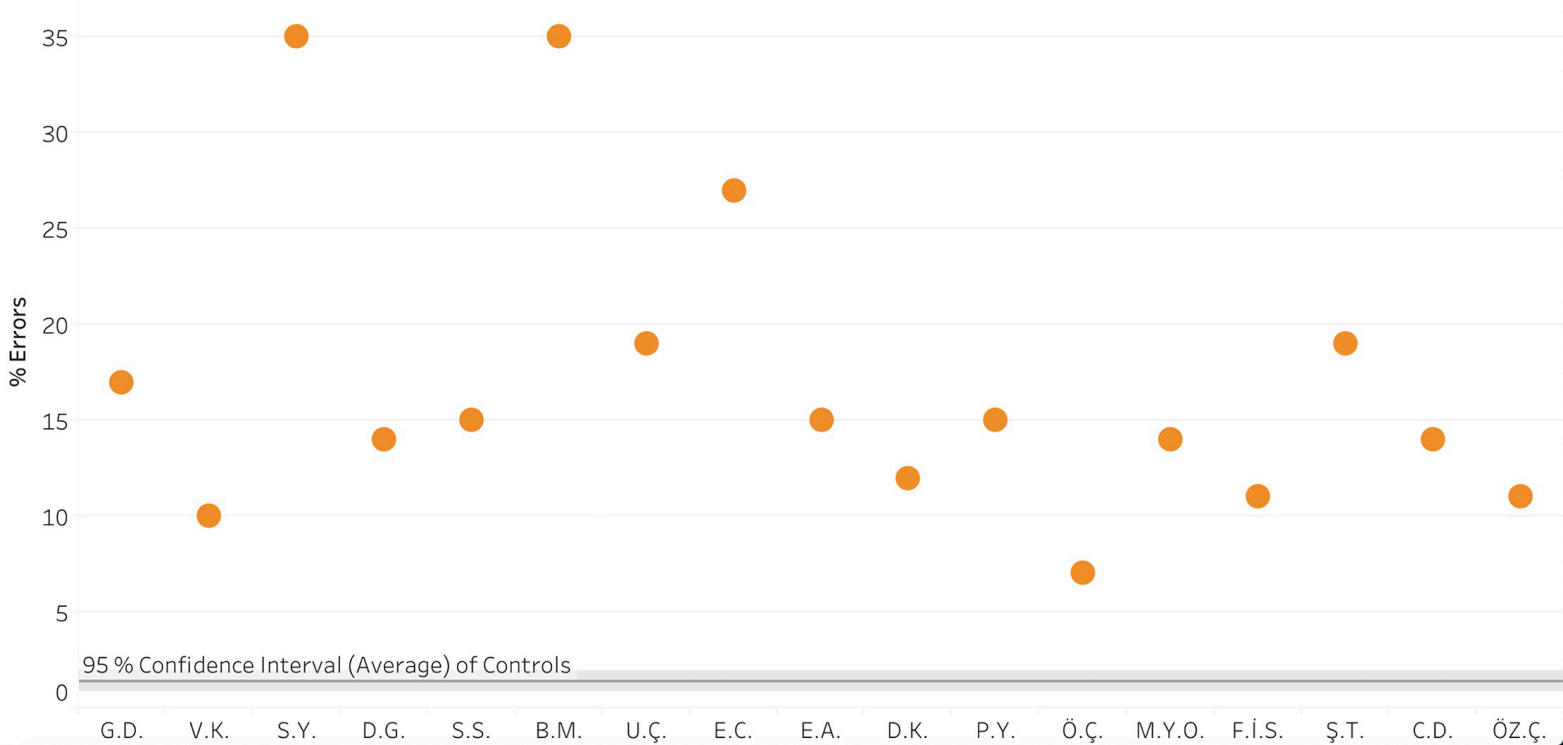
Reading words: %vowel errors (of the 124 words) for each vowel dyslexia participant (orange dots) compared to the control group (average in the horizontal line, with 95% confidence interval around it).

*Comparison of vowel letter errors of the dyslexic and control participants*. Fig 2 summarizes the vowel error rates of the 17 children with vowel dyslexia in word reading in comparison to the control group. Each of the 17 participants with vowel dyslexia made significantly more vowel errors than the control group (for each participant, *t*(59) > 4.04, *p* < .0001, Crawford & Howell’s [42], t-test for the comparison of an individual to a control group). The difference was also significant at the group level, where the vowel dyslexia group made significantly more vowel errors (17%, *SD* = 8%) than the control group (who made only 2.0% vowel errors on this list, *SD* = 1.7), Welch’s *t*(16) = 7.71, *p* < .0001, with a very large effect size (Hedges’ *g* = 3.9).

*Vowel errors vs*. *consonant errors*. The participants made far more vowel errors than consonant errors in their word reading. At the individual level, 14 of the 17 vowel dyslexia participants made significantly more vowel errors than consonant errors. This difference was also significant at the group level (*Wilcoxon z* = 3.60, *p* = .0002, *g* = 2.2). Table 3 summarizes the percentage of vowel letter errors out of the 124 words that had a lexical potential for a vowel error, and of consonant errors out of the 99 words that had a lexical potential for a consonant error.

**Table 3:** Percentage of vowel errors and consonant errors in reading words in the ÜZÜM word test.

Participant	% vowel errors	% Consonant errors	Significance
G.D.	17	9	χ^2^ = 2.91, *p* = .09
V.K.	10	5	χ^2^ = 2.19, *p* = .14
S.Y.	35	5	χ^2^ = 28.61, *p* = .0001
D.G.	14	7	χ^2^ = 2.53, *p* = .11
S.S.	15	3	χ^2^ = 8.51, *p* = .004
B.M.	35	9	χ^2^ = 20.16, *p* < .001
U.Ç.	19	5	χ^2^ = 9.13, *p =* .003
E.C.	27	10	χ^2^ = 10.42, *p =* .001
E.A.	15	5	χ^2^ = 5.33, *p =* .02
D.K.	12	1	χ^2^ = 10.16, *p* = .001
P.Y.	15	2	χ^2^ = 10.53, *p* = .001
Ö.Ç.	7	2	χ^2^ = 5.02, *p* = .03
M.Y.O.	14	3	χ^2^ = 7.69, *p* = .006
F.İ.S.	11	2	χ^2^ = 7.1, *p* = .008
C.D.	14	5	χ^2^ = 4.64, *p* = .03
Ş.T.	19	1	χ^2^ = 17.63, *p* < .0001
ÖZ.Ç.	11	0	χ^2^ = 11.93, *p* = .0006
**M (*SD*)**	**17 (8)**	**4 (3)**	***z* = 3.60, *p* = .0002**
Controls (*SD*)	2 (1)	2 (2)	

Another evidence for intact conversion of consonants is that the conversion of more complex consonantal graphemes—geminates—was also not impaired. Larsen et al. [46] suggested that a possible basis for vowel dyslexia is the spoken similarity of vowels. They proposed that "The more limited discriminability between vowels may result in less distinct memories for vowels, which in turn could leave more room for error during vowel GPC acquisition". If this is the case, the low discriminability between single and geminated consonants should be challenging too for the same participants.

We, therefore, analyzed the most sensitive type of stimuli: words that include geminates that have a non-geminate counterpart (i.e., reading the geminate as a single rather than a doubled consonant yields another existing word). For example, if the geminate in the target word *tekke* (lodge), is incorrectly read as a single consonant, this yields the existing word *teke* (goat). There were 15 such words in our reading aloud tests (4 in the word screening test, 11 in the NANE test described below in Section 3.4.3).

The results showed that even though the participants with vowel dyslexia made errors in converting vowels, they did not make errors in geminate consonants. The participants with vowel dyslexia made 5% geminate-to-single consonant errors (*SD* = 7%), which was within the range of the control participants (*M* = 2%, *SD* = 7%), Welch’s *t*(25) = 1.56, *p =* .13.

Namely, even though geminate consonants are quite similar to their counterpart single consonants, their conversion was unimpaired, which renders the idea that vowels are affected in vowel dyslexia due to their similarity less plausible.

### 3.3. Oral reading of nonwords

#### 3.3.1. Experimental stimuli: Nonword reading

The list included 52 nonwords, which were 4-to-6 letters long (*M* = 4.8 letters, *SD* = 0.7). All these nonwords were such that at least one vowel letter error creates an existing word (e.g., a vowel letter error in the nonword “kerük” can create the words “kürek”, “körük”, see Table 2 for examples), and 38 of these nonwords had also a potential for at least one consonant letter error that creates an existing word. All the items in the list were nonwords, and the participants were told before they started reading that this is a list of words that we invented, that do not exist in Turkish.

#### 3.3.2 Results: Nonword reading

**Fig 3:**
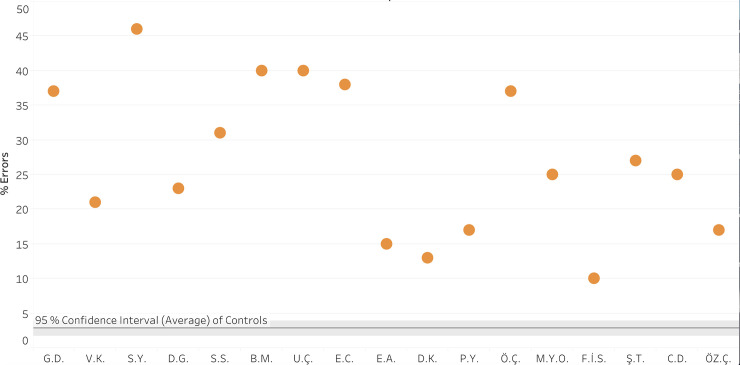
Reading nonwords: % vowel errors of each participant with vowel dyslexia (orange dots) compared to the control group average (horizontal grey line, and 95% CI around it).

*Comparison of vowel errors of the dyslexic and control participants*. The 17 participants with vowel dyslexia made an average of 27% (*SD* = 11%) vowel letter errors when reading the nonwords, a rate that is significantly higher than that of the control group (4%, *SD* = 4%), Welch’s *t*(17) = 8.46, *p* < .0001, *g* = 3.7. On the individual level (Fig 3), all the participants with vowel dyslexia made more vowel errors than the control group, for 16 of them it was significant (for each participant, *t*(59) > 3.37, *p’s* < .006, using Crawford & Howell’s [42], *t*-test), and for one it was marginally significant (*t*(59) = 1.45, *p* = .07).

**Fig 4:**
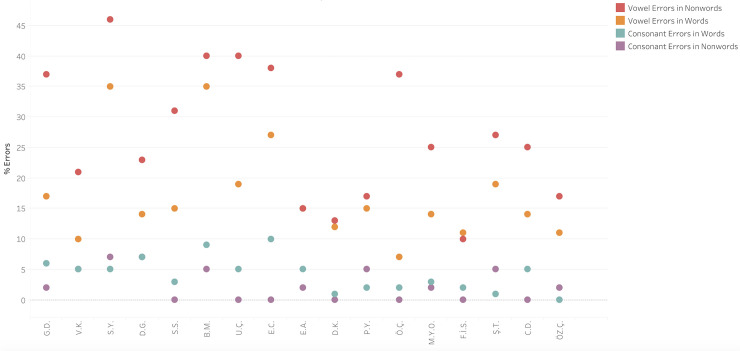
Percentage vowel letter errors (red and orange) and consonant letter errors (turquoise and purple) in reading words and nonwords per participant.

*Vowel errors vs*. *consonant errors*. We compared the rate of vowel errors that the participants made in reading the 52 nonwords from the ÜZÜM test that had a lexical potential for a vowel error and the 38 nonwords that had a potential for a consonant error. The results, summarized in Fig 4 and Table 4, clearly indicated that the participants with vowel dyslexia made significantly more vowel errors than consonant errors also in nonword reading. At the individual level, all participants with vowel dyslexia made more vowel errors than consonant errors, for 16 of them it was significant, and for one it was marginally significant (see Table 4 for individual *p’s*). This difference was also significant at the group level (*Wilcoxon z* = 3.60, *p* = .0003, *g* = 11.5).

**Table 4:** Percentage of vowel and consonant errors in reading nonwords in the ÜZÜM nonword test.

Participant	Vowel	Consonant	Significance
G.D.	37	3	χ^2^ = 14.6, *p* = .0001
V.K.	21	5	χ^2^ = 4.49, *p* = .03
S.Y.	46	8	χ^2^ = 15.3, *p =* .001
D.G.	23	8	χ^2^ = 3.64, *p* = .06
S.S.	31	0	χ^2^ = 14.22, *p* = .0002
B.M.	40	5	χ^2^ = 14.24, *p* = .0002
U.Ç.	40	0	χ^2^ = 20.02, *p =* .0001
E.C.	38	0	χ^2^ = 18.79, *p =* .001
E.A.	15	3	χ^2^ = 3.97, *p* = .05
D.K.	13	0	χ^2^ = 5.55, *p* = .02
P.Y.	17	5	χ^2^ = 4.79, *p* = .03
Ö.Ç.	37	0	χ^2^ = 17.6, *p* = .001
M.Y.O.	25	3	χ^2^ = 8.36, *p* = .004
F.İ.S.	10	0	χ^2^ = 3.87, *p* = .05
C.D.	25	0	χ^2^ = 11.1, *p* = .0009
Ş.T.	27	5	χ^2^ = 7.05, *p* = .008
ÖZ.Ç.	17	3	χ^2^ = 4.79, *p* = .03
**M (*SD*)**	**27(11)**	**3(3)**	
Controls (*SD*)	4 (4)	2 (4)	

*Note*. Shaded cells indicate a significant difference between vowel and consonant errors.

### 3.4. A sublexical deficit: More vowel errors in nonwords

If the deficit that gives rise to vowel dyslexia is in the sublexical route, then we expect to see more vowel errors in reading nonwords, which are read via the sublexical route, than in reading words, which can be read via the lexical route.

#### 3.4.1. Group-level results: more vowel errors on nonwords than on words

The participants with vowel dyslexia made significantly more vowel errors when they read nonwords (*M* = 27%, *SD* = 11%) than when they read words (*M* = 17%, *SD* = 8%), *Wilcoxon z* = 3.46, *p* = .0003, *g* = 1.1.

#### 3.4.2. Individual-level analysis and the interaction of vowel dyslexia with surface dyslexia

The individual-level analysis yielded an interesting pattern: all the 17 participants with vowel dyslexia showed impaired reading of nonwords. But whereas eight of the participants showed significantly fewer vowel errors in reading words compared to nonwords, other participants made many vowel errors on words as well (Table 5).

**Table 5:** Percentage of vowel errors in oral reading of words and nonwords in the ÜZÜM tests.

Participant	Words	Nonwords	Significance
**G.D.**	17	37	χ^2^ = 8.02, *p* = .005
**V.K.**	10	21	χ^2^ = 3.54, *p* = .05
**S.Y.**	35	46	χ^2^ = 2.05, *p =* .15
**D.G.**	14	23	χ^2^ = 2.34, *p* = .13
**S.S.**	15	31	χ^2^ = 6.21, *p* = .01
**B.M.**	35	40	χ^2^ = 0.52, *p* = .47
**U.Ç.**	19	40	χ^2^ = 9.32, *p =* .002
**E.C.**	27	38	χ^2^ = 2.10, *p =* .15
**E.A.**	15	15	χ^2^ = 0.02, *p* = .88
**D.K.**	12	13	χ^2^ = 0.06, *p* = .80
**P.Y.**	15	17	χ^2^ = 0.22, *p* = .64
**Ö.Ç.**	7	37	χ^2^ = 23.50, *p* = .0001
**M.Y.O.**	14	25	χ^2^ = 3.30, *p* = .70
**F.İ.S.**	11	10	χ^2^ = 0.11, *p* = .74
**C.D.**	14	25	χ^2^ = 11.10, *p* = .0009
**Ş.T.**	19	27	χ^2^ = 7.05, *p* = .008
**ÖZ.Ç.**	11	17	χ^2^ = 4.79, *p* = .03
**M (*SD*)**	**17 (8)**	**27(11)**	
**Controls(*SD*)**	2 (2)	4 (2)	

*Note*. Shaded cells indicate a significant difference between vowel errors in words and nonwords.

If the deficit leading to vowel errors in vowel dyslexia is in a vowel component in the sublexical route, then we expect vowel errors to occur only when a person reads via the sublexical route. Hence, it would be important to examine whether the children who showed similar vowel error rates in words and nonwords had indications that they were, in fact, reading words via the sublexical route.

#### 3.4.3. Testing surface dyslexia

To examine which of the participants with vowel dyslexia were reading words via the sublexical route, i.e., whether they had surface dyslexia, we assessed their reading of irregular words. We tested nine of them in a surface dyslexia task (NANE test), and also analysed for all of them the reading of 17 words sensitive to surface dyslexia in the screening task and in the vowel dyslexia task. (Because there were only 17 such words in the screening+vowel tasks, in the surface dyslexia analysis we included children who did not take the surface dyslexia test only if they were clearly above or below the normal threshold of regularization errors on these 17 irregular words, i.e., had 0 regularization errors or 3 and above. This yielded, for this analysis, 14 children: 5 children who read only the 17 irregular words, in addition to the 9 who took the surface dyslexia test.)

*NANE*: *The surface dyslexia task*. The surface dyslexia task from the *FRİGÜ* dyslexia battery [41] contains 51 4-to-8 letter irregular words (*M* = 5.3, *SD* = 1). Even though Turkish has a very transparent orthography, some words, mainly loanwords, are irregular and cannot be correctly converted when reading solely through the sublexical route. The main type of irregularity came from loanwords from Farsi and Arabic, in which a vowel is pronounced as a long vowel, but written with a single vowel, which regularly corresponds in Turkish to a short vowel. An example is the word *katip*, which means, like in the original Arabic, a writer. In Arabic, it is written with a single vowel letter, which stands for a long vowel (as only long vowels are represented as vowel letters in Arabic), and once borrowed into Turkish, it was also written with a single vowel letter but pronounced as a long vowel, which, in Turkish is regularly written with a double vowel letter. Another type of irregularity in loanwords occurs in words that originally included phonemes that are not part of the Turkish phonological inventory. For example, The Turkish word *nane* (mint), was also borrowed from Arabic. But the original Arabic word is pronounced with a glottal consonant, *Naʔnaʔ*; the glottal consonant is not part of the Turkish phonological inventory and it is instead pronounced as a vowel /naane/. So this word, too, is pronounced with a long vowel (*naane*) but written with a single vowel (*nane*). Reading these words via the sublexical route may result in reading them with a short, rather than long, vowel. Out of the many loanwords, we selected the ones that had a higher frequency, and a higher chance that children will be familiar with them (mean frequency of the words in the Nane test was between 561 and 84656 per million (mean 9660, *SD* = 18928, and their mean dispersion was 0.86, *SD* = 0.07). Because vowel length differences are subtle, we used extra caution with coding vowel length errors, and in the irregular words had three independent coders listen and code the responses to these words.

The results showed a very tight relation between whether or not a child had surface dyslexia and whether or not they made significantly more vowel errors in reading nonwords compared to words: 8 children had no surface dyslexia (i.e., their rate of regularization errors was within the normal range), and 5 children had surface dyslexia (i.e., they made significantly more regularization errors on the irregular words compared to the controls using Crawford and Howell’s [42] t-test). One child had a marginal rate of regularization errors. (Notice that it is unlikely that the errors on these irregular words were a result of vowel dyslexia rather than of surface dyslexia because most of the children with vowel dyslexia did not make such surface errors).

The important finding, shown in Table 6, was that all 8 children who did not have surface dyslexia and were able to read words via the lexical route, made significantly more vowel errors on nonwords compared to words. In contrast, 4 of the 5 children who had surface dyslexia in addition to their vowel dyslexia did not show a significant difference between vowel errors in words and nonwords. This finding supports the idea that vowel dyslexia errors emerge when the person reads via the sublexical route. (Note, that the reason the children who did not make more surface errors than the age-matched controls still made some vowel errors in words is that they were still reading some words via the sublexical route. As fourth-graders, they might not have already completely filled their lexicons. Hence, they still had to read words that are not in their lexicons yet, via the sublexical route. This is supported by the word frequency effect according to which lower frequency words were read with more vowel errors, suggesting that some, less-frequent, words were read via the sublexical route, probably because they did not have stable lexical representations yet. Still, they made significantly more vowel errors on nonwords, which were all read via the sublexical route.)

**Table 6:** Percentage of vowel errors in words compared to nonwords (out of the ÜZÜM test and the words with lexical potentials for vowel errors in the FRİGÜ screening test), and their relations to the rate of surface errors in irregular words (out of the NANE test and the irregular words in the FRİGÜ screening test).

	%Vowel errors			% Surface errors	
Participant	Words (*N* = 224)	Nonwords (*N* = 81)	Significance	Screening test (*N* = 17)	Surface test (*N* = 51)
**No surface dyslexia**					
**G.D.**	14	31	χ^2^ = 10.76, *p* = .001	0	-
**V.K.**	7	22	χ^2^ = 14.86, *p* = .0001	6	8
**D.G.**	12	21	χ^2^ = 4.32, *p* = .04	0	2
**S.S.**	13	35	χ^2^ = 18.30, *p* = .0001	12	8
**B.M.**	29	52	χ^2^ = 13.04, *p* = .0003	0	12
**Ö.Ç.**	6	23	χ^2^ = 18.25, *p* = .0001	6	6
**Ş.T.**	13	25	χ^2^ = 5.54, *p* = .02	6	2
**ÖZ.Ç.**	8	17	χ^2^ = 4.78, *p* = .03	0	-
**Marginal**					
**U.Ç.**	14	36	χ^2^ = 18.16, *p =* .0001	18	14
**Surface dyslexia**					
**S.Y.**	28	35	χ^2^ = 1.36, *p =* .24	24	43
**C.D.**	9	19	χ^2^ = 4.78, *p =* .03	24	-
**D.K.**	9	15	χ^2^ = 1.82, *p* = .18	24	-
**P.Y.**	9	17	χ^2^ = 4.19, *p* = .06	18	-
**F.İ.S.**	8	12	χ^2^ = 1.33, *p* = .25	12	25
**Threshold for surface dyslexia from the control group**				12	16

#### 3.4.4. The interaction of vowel dyslexia with surface dyslexia

To further examine whether indeed vowel dyslexia results from a deficit in the sublexical route, we calculated the correlation between *p*_vowel error in words_ and *p*_vowel error in sublex_* *p*_reading via sublex_. Namely, we calculated the probability to make a vowel error when reading existing words (estimated by the participant’s percentage of vowel errors in reading words). We correlated this rate with the multiplication of two probabilities: the probability of each participant to make a vowel error when reading via the sublexical route (estimated according to the percentage of vowel errors they made when reading nonwords) and the probability that the participant would read an existing word via the sublexical route (estimated by the rate of surface errors in reading words, indicating reading words via the sublexical route). If indeed vowel dyslexia results from a vowel-specific deficit in the sublexical route, these two should correlate. (Namely, people who make vowel errors in nonwords, and who read words via the sublexical route are expected to make vowel errors in words. If one of these probabilities is zero, no vowel errors are expected in reading existing words: i.e., even if reading predominantly happens via the sublexical route, if the vowel processing in the sublexical route is intact, with a zero *p*_vowel error in sublex_, the participant is not expected to make vowel errors in words).

The correlation between the vowel error rate of our participants (*n* = 14, see Table 6) in reading words and the multiplication of the two other probabilities was very high, *r*(12) = .72, *p =* .003. These results are in line with the high correlation that was found between these probabilities in vowel dyslexia in Hebrew [20].

#### 3.4.5. Nonlexical responses: Supporting evidence for sublexical impairment

To further examine our conclusion that the deficit of our participants with vowel dyslexia is in the sublexical route, we investigated the lexicality of their responses in reading words and nonwords. Our prediction is that if their deficit is in the sublexical route, they are expected to produce nonlexical error responses.

When the participants with vowel dyslexia made a vowel error, most of their responses were lexical. However, still, a third of their vowel error responses were nonlexical, both in reading words, where 36% of their error responses were nonlexical (*SD* = 13%) and in reading nonwords, where 35% of their error responses were nonlexical (*SD* = 16%), see Table 7 for individual rates of nonlexical responses. This finding supports the assumption that the participants with vowel dyslexia make their vowel errors while reading via the sublexical route.

**Table 7:** Percentage of nonlexical responses out of the responses with vowel letter errors in reading the 124 words and 52 nonwords in the ÜZÜM tests.

Participant	Words	Nonwords
**G.D.**	43	47
**V.K.**	62	45
**S.Y.**	51	58
**D.G.**	53	33
**S.S.**	22	25
**B.M.**	42	48
**U.Ç.**	35	19
**E.C.**	35	25
**E.A.**	28	38
**D.K.**	40	29
**P.Y.**	44	22
**Ö.Ç.**	33	47
**M.Y.O.**	18	23
**F.İ.S.**	29	40
**C.D.**	29	0
**Ş.T.**	39	29
**ÖZ.Ç.**	7	67
***M* (*SD*)**	**36 (13)**	**35 (16)**

The finding that the participants provided nonlexical responses on as much as a third of their erroneous responses is especially remarkable given the strong lexical biases built into the properties of the nonwords we used, as well as the architecture of the reading system. Firstly, all nonwords in the ÜZÜM nonword test were constructed in a way that a vowel error would create at least one other existing word (with an average of 2.9 other existing words resulting from vowel errors, SD = 1.1). The probability for lexical responses is also enhanced by the architecture of the reading process. The output of the sublexical route flows to the phonological output buffer, which receives feedback from the phonological output lexicon. The phonological output lexicon provides support from lexical entries in the phonological output lexicon to the sequence of phonemes held in the phonological output buffer. When the sequence is ambiguous or underspecified with respect to vowels, the lexical support causes the production of a lexical sequence that matches the partial information. Given this, we reasoned that if even in these conditions of strong lexical bias the participants still produced nonlexical responses, in a third of their responses, this supports the conclusion that the source of the vowel errors of our participants was a deficit in the sublexical route.

#### 3.4.6. Interim summary: A deficit in the sublexical route

The results presented in this section, showing more vowel errors when the participant is reading via the sublexical route: more vowel errors on nonwords than on words; more vowel errors when a participant is reading via the sublexical route because of surface dyslexia or because of a tendency to read aloud; and the production of nonlexical responses, indicate that the source of the vowel dyslexia of our participants was a vowel-specific deficit in the sublexical route.

### 3.5. Where is the deficit in the sublexical route?

The results thus far suggest that a deficit in the sublexical route is responsible for the vowel dyslexia of our participants. But what exactly is impaired in the sublexical route? Is it the conversion itself or rather a buffer in the sublexical route that specializes in holding the vowels until the completion of conversion? To examine the exact locus of the deficit in the sublexical route, we conducted several analyses on vowel error types and effects on reading.

#### 3.5.1. Single vowel letter conversion: The deficit is not in the conversion itself

To examine the participants’ ability to convert vowel letters to phonemes, we asked 13 of the 17 participants with vowel dyslexia to sound out single letters. All Turkish letters were presented as single letters on a white sheet of paper, in random order. These letters included the 8 vowel letters.

The results were that all the participants with vowel dyslexia sounded out correctly all the vowel letters presented to them, suggesting that their ability to convert single vowel letters to phonemes is intact and the deficit is present only when the vowel letters are incorporated in a nonword (or a word) so that several vowels need to be processed together in the sublexical route. This conclusion is also supported by their very low error rate in reading words with a single vowel letter, as described below in Section 3.5.3.

#### 3.5.2. Vowel error types: Not limited to substitutions

Had the deficit in the sublexical route been a deficit in vowel letter conversion, we would expect to see mainly vowel substitutions (and possibly also omissions). Therefore, we examined the types of vowel errors that the participants produced.

We found that the participants made not only vowel substitutions, but also other types of vowel identity errors: omissions and additions of vowel letters, as well as vowel migrations, as exemplified in Table 2 (for a more extensive list of examples for errors that the participants made, see S3 Table).

We calculated the number of vowel errors of each type out of the number of words in which such an error created an existing word. We did so because not all words allowed for all vowel error types to create other existing words, and because the participants made more vowel errors on words in which a vowel error creates an existing word. (In the screening and üzüm tests, the participants made significantly more vowel errors on words in which a vowel error creates existing words, *M* = 12.3%, *SD* = 6.1%, than on words in which no vowel error creates another existing word, *M* = 4.4%, *SD* = 5.6%, *Wilcoxon z* = 3.60, *p* = .001, *g* = 1.31).

**Fig 5:**
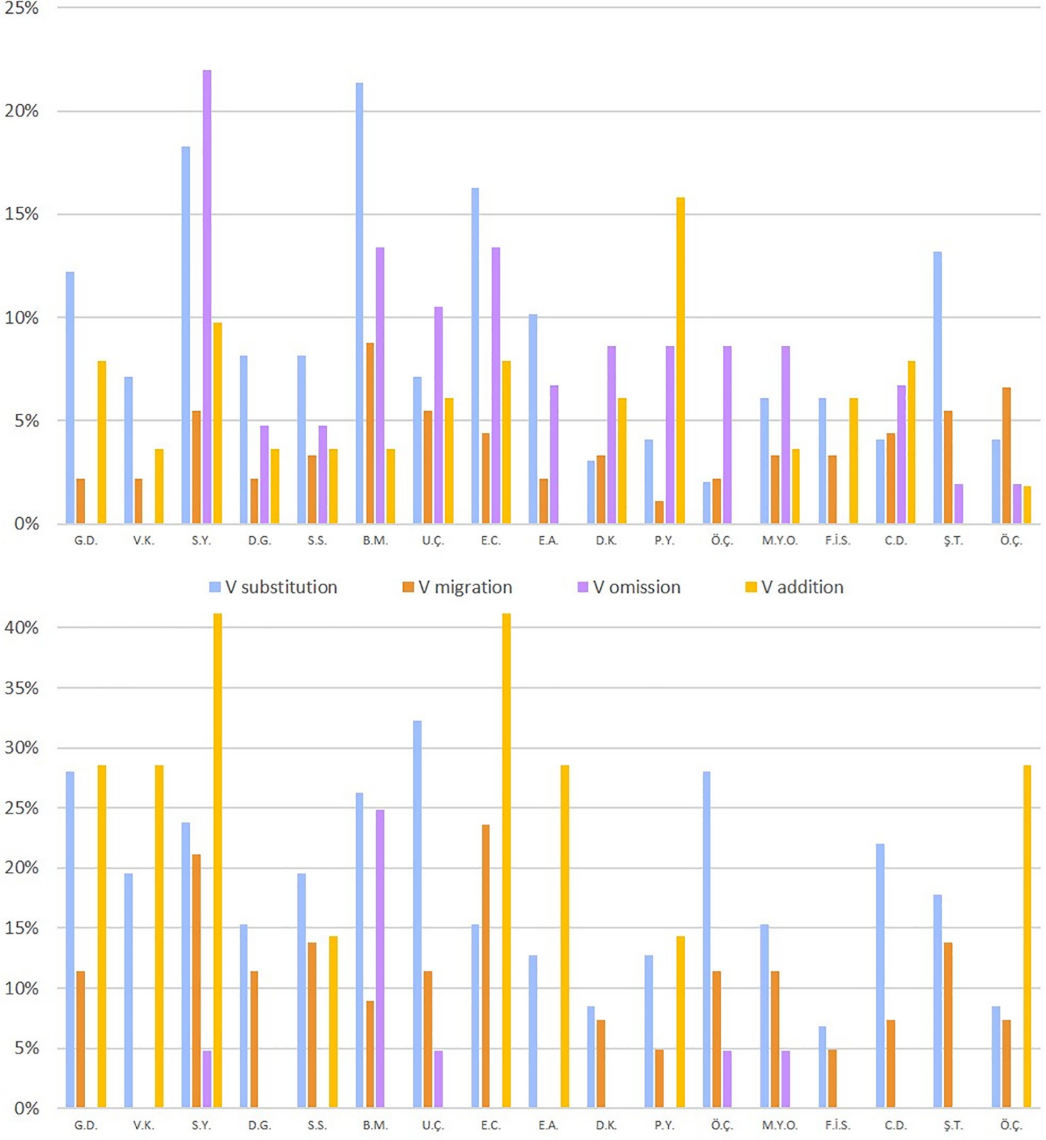
Distribution of vowel error types in words per participant out of the target words(top) and in nonwords(bottom)per participant out of the target nonwords with a lexical potential for such errors.

This analysis yielded a considerable individual variation with respect to the distribution of the various error types, as shown in Fig 5. At the group level, the distribution of vowel error rates (average percentage errors of each kind out of the number of words that allow for this error to create a word) was, for nonwords: 18% substitutions, 10% migrations, 3% omissions, and 13% additions; for words: 9%, 4%, 7%, and 5%, respectively.

The finding that the participants did not make only vowel substitutions (and omissions) but also made many vowel migrations and vowel additions supports the idea that the deficit leading to vowel dyslexia is not in the conversion itself, but rather possibly in a sublexical component that holds several vowels together.

#### 3.5.3. Effect of number of vowel letters: Evidence for a vowel-buffer deficit

If the deficit is in a sublexical component that holds several vowels together, a vowel buffer of a kind, we would expect to see the hallmark of buffer impairments: a length effect. We examined the effect of the number of vowel letters in the word on the rate of vowel letter errors. The first analysis was a comparison of vowel error rates in 17 words that contain a single vowel, 92 words that contain 2 vowels, and 32 words that contain 3 vowels (and one word with 4 vowels). The participants with vowel dyslexia made 2% vowel errors in words that contain a single vowel letter (SD = 5%), 14% (*SD* = 8%) vowel errors in 2-vowel words, and almost twice as many vowel errors (*M* = 26%, *SD* = 13%) in words that contain 3(-4) vowels. These results indicated a significant effect of number of vowel letters in the word. Importantly, this length effect far exceeds the effect expected by a per-vowel-letter length effect: if we take the 1-vowel words to estimate the probability of a vowel error per-vowel-letter, this would be around 2%, then the per-vowel-letter probability for 2-vowel-letter words should be 4%, and the 3-vowel-letter words should have caused around 6% errors. Instead, 2-vowel words showed 3.5 times more errors than expected by the per-vowel-letter probability, and 3-letter words show 4.5 times more vowel errors than expected. This suggests a buffer effect on the holding of several vowel letters together.

**Fig 6:**
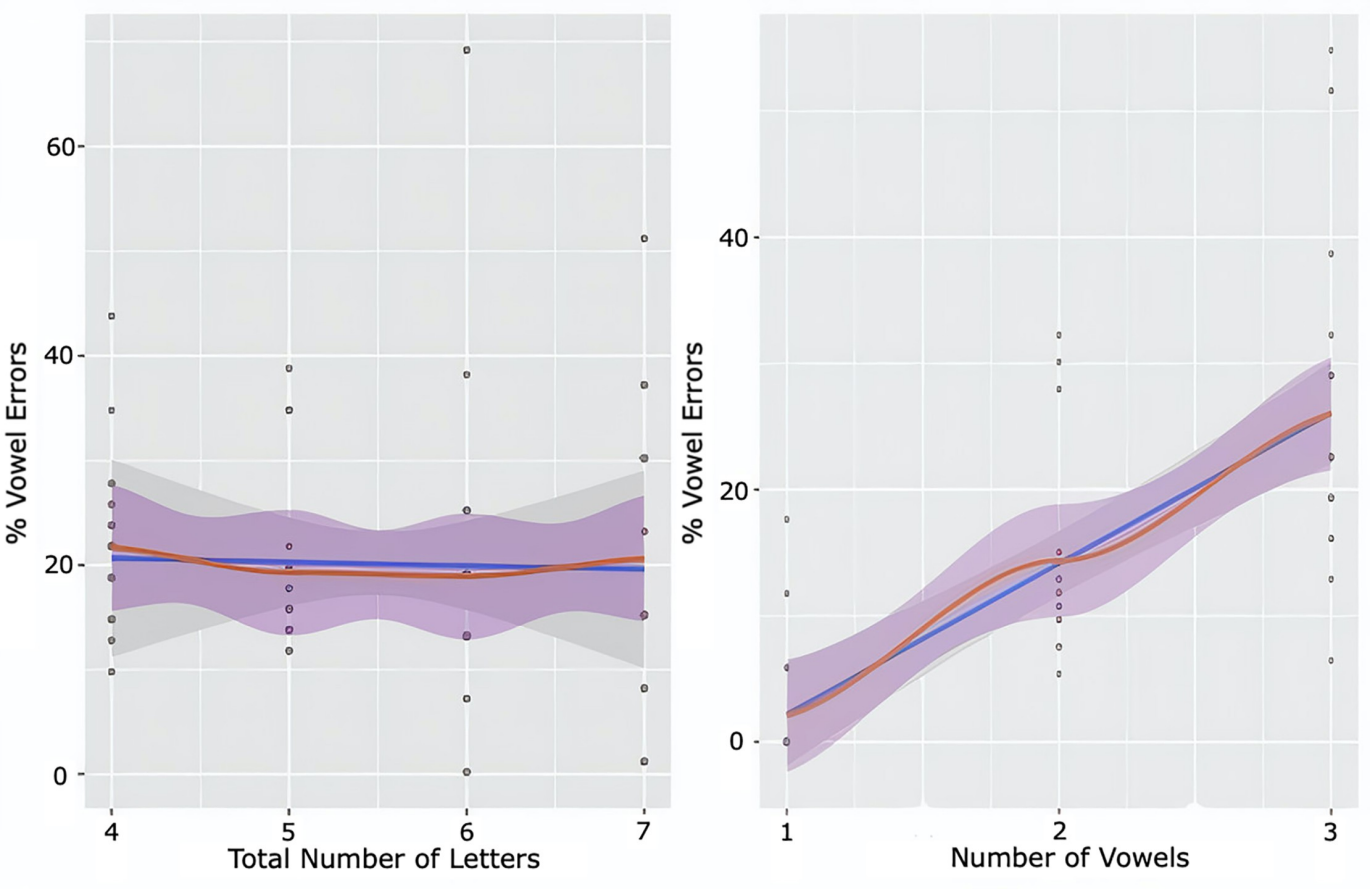
The regression lines (blue) and LOWESS smoothing curve (red) showing the relation between vowel error rate and the number of vowels in the word (left) and between vowel error rate and the total number of letters in the word (right).

We also conducted regression analyses for the effects of the number of vowels and of the number of letters in a word on vowel error rates. The results, summarized in Fig 6 showed that whereas the number of vowels had a critical effect on the error rate (F(1, 49) = 58.87, *p* < .0001, R2 = .53), the total number of letters in the word did not have a unique contribution to the rate of vowel errors (F(1, 66) = 2.74, *p* = .10, R2 = .03). These results lend considerable support for the notion of a separate buffer for vowels in the sublexical route, which may be the source of the vowel letter dyslexia of our participants.

#### 3.5.4. Words with consistent vowels and words with different vowels

If the vowel deficit of our participants is indeed related to a limited vowel-specific buffer in the sublexical route, then vowels that are held together in this buffer may affect one another. If each vowel is processed separately in the sublexical route, then no effect is expected for the other vowels in the word. We examined whether words with different vowels (e.g., *çilek*, *kıran*) are more challenging than words in which all vowels are the same (*ayran*, *araba*, *geveze)*. We compared the rate of vowel errors that occurred in 2–3 vowel words in which all vowels were the same to the rate of vowel errors the same participants made in words that contained at least two different vowels.

The results of this analysis indicated that the participants indeed made significantly more vowel errors in words in which there were at least two different vowels (*M* = 17.8%, *SD* = 8.4%) than on words (with at least 2 vowels) in which all vowels were the same (*M* = 9.6%, *SD* = 14.4%), *Wilcoxon z* = 2.51, *p* = .01, *g* = 0.8.

An analysis of specific vowel error types in 2–3 vowel words in which all the vowels were the same and in words in which there were at least two different vowels yielded the pattern summarized in Table 8. The participants made more vowel substitutions and more vowel omissions in words with different vowels than in words with the same vowels, and about half of the substitutions in words with different vowels were substitutions with another vowel that appeared elsewhere in the word. Vowel additions showed the opposite pattern, with more vowel additions in words with the same vowel.

**Table 8:** Percentage of different types of vowel errors in words with more than one vowel: Words with different vowels compared with words in which all vowels are the same.

	Different vowels	All vowels are the same
Vowel substitution	4.1%	1.3%
with a vowel outside the word	2.4%	1.3%
with another vowel in the word	1.7%	-
Vowel omission	1.0%	0.2%
of a vowel that appears only once in the word	0.9%	0.2%
of a vowel that appears more than once in the word	0.1%	0.0%
Vowel addition	1.1%	1.9%
of a vowel that does not appear in the word	0.6%	1.1%
of a vowel that appears in the word	0.5%	0.8%
Vowel migrations	1.1%	0.2%

#### 3.5.5. Interim summary: A deficit in a vowel buffer in the sublexical route

The results presented in this section indicate that the deficit in the sublexical route is not in the conversion itself–as indicated by the intact conversion of single letters and by the existence of vowel migration and addition errors, but rather in a vowel-specific buffer in the sublexical route. This conclusion is supported by the existence of vowel migration and addition (and omission) errors; by the pronounced vowel letter length effect whereby the more vowels there are in a word, the more this word is susceptible to vowel error; and the effect of the existence of repeating vowels within the word.

The finding that vowel errors in nonwords created lexical responses in two-thirds of the responses also sheds some light on the nature of the vowel deficit. It suggests that the vowel deficit in the sublexical route causes underspecification of the vowels in the target words, so that when this partial information arrives in the phonological output buffer, the lexical support from the phonological output lexicon causes the production of an existing word that matches this partial information. Had the deficit been characterized by incorrect conversion, we would not expect such a tendency for lexicalization.

Given the underspecification of vowels, additional factors such as vowel frequency and vowel harmony may affect the responses that are eventually produced, as we examine in the next section.

### 3.6. Characteristics of vowel errors and what affects responses when the sublexical vowel buffer fails

#### 3.6.1. An effect of vowel frequency on the error response

We examined whether the frequency of the vowels affected the vowel errors of the participants. To do so, we examined the effect of frequency on various error types. For addition errors, we examined whether the vowels added were of high frequency. For substitution errors, we tested whether the substituting vowels were the higher frequency vowels, and we also examined the relative frequency of the target and substituting vowels: for each substitution error we tested whether the vowel substituting the target vowel was of higher frequency than the target vowel. For these analyses, we used the vowel letter frequency database [47].

The results of this analysis indicated that the frequency of the vowels played a major role in the vowel errors produced by our participants: 75% of the vowel additions were of the two most frequent vowels (a and e), 55% of them were the most frequent vowel, *a*.

Vowel substitutions showed a very similar effect of vowel frequency: Out of 112 words in which a vowel was substituted, 47 of the substituting vowels were *a*, the most frequent vowel, 26 were *i*, the third most frequent vowel, 20 were *e*, the second most frequent vowel, and the rest 19 distributed between all the rest 5 vowels (*ı*, *o*, *u*, *ü*, *ö*). The majority of vowel substitutions were substitutions of a lower-frequency vowel with a higher frequency vowel. When comparing substitutions of the 5 lower-frequency vowels and the 3 higher frequency vowels (a,e,i), a clear tendency to substitute a lower-frequency vowel with a higher-frequency one was observed: All 43 substitutions of one of the 5 lower-frequency vowels were substitutions with one of the 3 higher-frequency vowels. The 3 higher-frequency vowels were also substituted mainly with one of the other high-frequency vowels (51 of the 69 substitutions). So there were significantly more substitutions from 5 lower frequency vowels to the 3 higher frequency vowels than vice versa, χ^2^ = 58.35, *p <* .0001.

This suggests that when the conversion of vowels in the sublexical route fails, the reader falls back on vowel frequency and uses the more frequent vowels.

#### 3.6.2. An effect of vowel harmony on error responses

Vowel harmony (VH) in Turkish, as described in the Introduction, is a robust phenomenon, mastered by Turkish speakers, and acquired very early [22]. We were therefore interested to know whether this property affects vowel errors in individuals with vowel dyslexia.

*Do vowel errors conform to* vowel harmony *constraints*? To examine this question we performed two analyses. The first examines whether, once a person with vowel dyslexia makes a vowel error in the word, they tend to produce a harmonic error response, i.e., a response that conforms to vowel harmony constraints. We did so by examining, for each vowel error, whether it created a harmonic or non-harmonic response, separately for harmonic and non-harmonic target words and nonwords.

Here are examples for each type of error:

Harmonic target word with a harmonic error response: bakir → bakar or bıkar
Harmonic target word with a nonharmonic error response: bakar → bekar or bakir
Nonharmonic target word with a harmonic error response: kenar → kanar or kınar
Nonharmonic target word with a nonharmonic error response: hale → hela or hali

The participants had a very clear tendency, once they made a vowel error, to produce a response that conformed to the VH constraints. Of all the words and nonwords in the screening test and the vowel dyslexia test that included more than one vowel, 85% (187 of 221) of the vowel errors on harmonic target words and nonwords were harmonic, and 84% (16/19) of the vowel errors on non-harmonic target words and nonwords were also harmonic. It seems that once the participants are not sure what to do with the vowel letters in the sublexical route, they rely on general phonological rules in the language, like VH, to guide their phonological choice. The effect of vowel frequency reported above may be another aspect of the same tendency.

Interestingly, the few vowel errors that the typical readers made on these words showed the same tendency: 84% of the vowel errors they produced on harmonic target words created harmonic responses, and 83% of their vowel errors on nonharmonic target words created harmonic responses.

At the individual level, all of the children with vowel dyslexia showed the same clear pattern, they tend to harmonize the vast majority of the vowel errors they produce (*M* = 84% of the vowel errors, *SD* = 10%, range 67%−100%).

*Do vowel errors occur more frequently in target non-harmonic stimuli*? A second analysis examined whether vowel errors occur more frequently in target non-harmonic compared to harmonic stimuli.

In an analysis of all types of vowel errors together, there was no difference in the rate of vowel errors on harmonic and non-harmonic target stimuli (9.6% errors on harmonic words, 8.6% vowel errors on non-harmonic words; 14.9% errors on harmonic nonwords; and 7.2% vowel errors on non-harmonic nonwords).

Even when we calculate only vowel substitution and omission (types of vowel errors that may "amend" nonharmonic target words), the picture remains the same, with 7.8% errors on harmonic words, and 7.5% vowel errors on non-harmonic words; 12.6% errors on harmonic nonwords, and 6.8% vowel errors on non-harmonic nonwords.

Namely, the vowel errors do not stem from the participant’s urge to harmonize non-harmonic words. However, once they are in a vowel reading uncertainty, they tend to follow VH constraints and produce harmonic responses. This may be an instance of the "linguistic constraints on reading" that Berent and Perfetti were discussing in their paper [48].

#### 3.6.3. Effect of lexical frequency

We also examined the effect of lexical frequency on vowel errors in children with vowel dyslexia. In line with the recommendations of, e.g., Giers [44], we used dispersion values for this analysis. We made two types of analysis. The first examined whether lower frequency words are more susceptible to vowel errors than higher frequency ones. The second examined the relative dispersion of the target word and the vowel error response.

The first analysis included 124 words (Mean dispersion = 0.84). We calculated the correlation between the total rate of vowel errors of the vowel dyslexia group on each word with the dispersion index of this word. The results yielded a small negative marginally significant correlation between frequency and vowel error rate, *Pearson r* (121) = -0.13, *p* = .07. It may be that words with higher frequency have a higher probability of being read via the lexical route and hence–without vowel errors, whereas lower frequency words may not exist in the reader’s lexicon and therefore would be read via the sublexical route, which would then give rise to a vowel error. Still, the effect was very small, if it was an effect at all.

The second analysis compared, for each of the target words on which a vowel error was made, the dispersion values of the target and the highest frequency word that was a result of a vowel error to this word. This analysis included 52 words from the vowel dyslexia word list on which at least one vowel error was made, and that had frequency data in the frequency database of Turkish [45]. This analysis indicated no effect of relative frequency between the target and the word resulting from the vowel error: the mean dispersion frequencies of the target words were exactly identical to those of the produced words, 0.88 (*SD* = 0.6), with no difference between the two, *t*(102) = 0.00, *p* = 1, suggesting that there was no tendency to read a higher frequency word instead of a lower frequency target. This makes sense if vowel errors result from reading via the sublexical route, which should not be sensitive to lexical effects such as word frequency.

Namely, whereas more frequent words stand a better chance to exist in the participants’ lexicons and hence to be read via the lexical route, and hence without vowel errors, once the word is read via the sublexical route, there is no effect of word frequency, just an effect of the frequency of its vowels, as we have seen in section 3.6.1.

### 3.7. Almost no vowel errors in morphological affixes: Evidence for a separate morphological route

According to some recent analyses [15] (see Fig 1), the sublexical route includes separate routes for the conversion of whole morphological affixes and for the conversion of stem graphemes. If this is indeed the case, then it would be interesting to examine whether the vowel deficit affects both sub-routes or only the grapheme-to-phoneme conversion route. And what better language in which to examine this than the morphologically rich Turkish.

To examine this question we compared vowel errors in stems and vowel errors in affixes, calculated out of the words in which such errors create other existing words. This analysis encompassed 120 words that allow for at least one vowel error in the stem (see example 1) and 55 words that have at least one potential for a vowel error in an affix (which results in a morphological substitution of one suffix with another, as in example 2).

(1)                biz-i                (us)                        >>                bez-i                    (the cloth)

              us-ACC                                                                            cloth-ACC

(2)                biz-e                (to us)                    >>                        biz-i                    (us)

                us-DAT                                                                            us-ACC

The results of this analysis yielded a sharp difference between vowel errors in morphological affixes and vowel errors in stems: the participants with vowel dyslexia made 15% vowel errors in stems (*SD* = 8%), and only 3% vowel errors in affixes (*SD* = 2%). Vowel errors occurred significantly more often in stems than in affixes, *Wilcoxon z* = 3.60, *p* = .0003, *g* = 1.3.

Additionally, unlike the stems, in which the participants made nonword errors on a third of the items (see Section 3.4.5), the participants never created a non-suffix when they made a vowel error on a suffix, they only substituted the suffix with another existing suffix.

Another indication for their good morphological abilities is that they used allomorphs correctly: The vowels of the suffix must harmonize with the vowels of the stem. In instances in which the children made a vowel error in the stem (e.g., the stem *kane* instead of the stem *kan*), they changed the suffix accordingly, so that it will meet the vowel harmony requirements (in this case, the child changed the suffix *lı*, matching the original vowel *a*, to its allomorph *li*, matching the vowel *e* he added, resulting in *kaneli* instead of *kanlı*). Namely, their preserved morpho-phonological knowledge about vowel harmony as a selector of the right allomorph enables them to apply this rule even when the stem changed in a way that it is no longer an existing word.

These results suggest that at the stage in which vowel errors occur, the morphological structure of the word is already available. They also indicate that the sublexical conversion of affix morphemes occurs separately from the stem graphemes, and probably as whole-morphemes, which are not decomposed to their constituent letters. Morphologically complex words are decomposed to stems and affixes pre-lexically [49], in the orthographic input buffer stage. The orthographic form of the (whole) affix is converted to an abstract phonological form of the whole affix. The abstract phonological affix arrives in the phonological output buffer, which applies the morpho-phonological rules of the language and selects the allomorph that matches the stem that eventually arrived in the buffer [15].

### 3.8. Silent reading: No vowel errors when the sublexical route is not involved

If the source of vowel letter errors is a deficit in the sublexical route, participants with vowel dyslexia should not make vowel errors when they perform tasks without using the sublexical route. To examine this, we assessed their reading in tasks that did not involve reading aloud: same-different decision, which requires only the orthographic-visual analyzer [50], and written word comprehension, which requires the orthographic-visual analyzer and buffer, the orthographic input lexicon, and the semantic lexicon, but not the sublexical route. (We had initially also included a lexical decision task, but it turned out that the instructions were too difficult to understand for all participants, including the controls, who made approximately 20% errors on this task, so we do not report this task here).

#### 3.8.1. Same-different decision

The participants were presented with a list of 101 pairs of 4–7 letter words and nonwords (*M* = 5.1 letters, *SD* = 0.7), printed on white pages. The pairs included 43 pairs of words that differed in a vowel letter, 29 pairs of nonwords that differed in a vowel letter, and 29 pairs of identical words, randomly ordered. We asked participants to circle the pairs that contain the same words, without reading aloud.

In marked contrast to their performance in the reading aloud tasks, the participants with vowel dyslexia performed well on the same-different task, with an average of 94.5% correct (*SD* = 3.2%). Their error rate in the same-different decision on nonwords, 5.5% (*SD* = 5%), was significantly lower than their vowel error rate on reading aloud of nonwords, 27.3% (*SD* = 11%), *Wilcoxon z* = 3.38, *p =* .0007, *g* = 2.5. The same was found for words: they made 5.3% (*SD* = 5%) errors in the same-different task, a significantly lower error rate than they had in reading aloud (which was 17%, *SD* = 8%). This also formed a significant difference, *Wilcoxon z* = 3.62, *p =* .0007, *g* = 1.6.

At the individual level, too, 12 of the 15 participants with vowel dyslexia performed better on nonwords in the same-different task than on the oral reading task (*χ*^*2*^ > = 4.27, *p* < .05), and 8 of the 15 participants with vowel dyslexia performed better on words in the same-different task than on the oral reading task (*χ*^*2*^ > = 4.06, *p* < .05).

The vowel dyslexia participants made 3% (*SD* = 8%) errors in which they said "same" for pairs of words differing in vowel letters, 6% (*SD* = 5%) errors in which they said "same" for pairs of nonwords differing in vowel letters, and 6% (*SD* = 5%) errors in which they said "different" for identical pairs.

**Fig 7:**
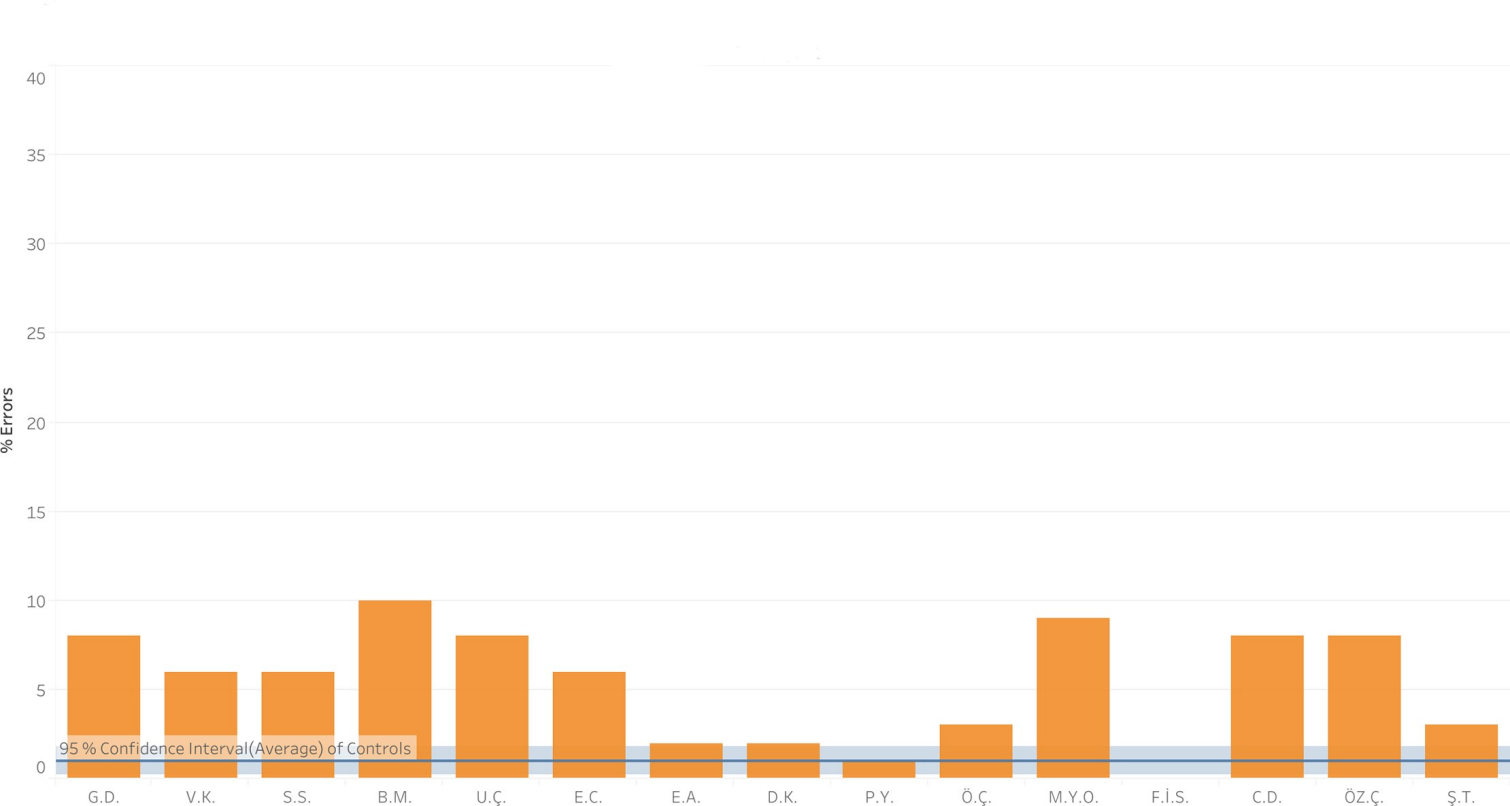
% errors of the participants with vowel dyslexia in the same-different task (% errors of 101 items) compared with controls (mean errors and 95% CI around it).

It is interesting to note that two of the participants with vowel dyslexia, DG and SY, insisted on reading aloud even though the instructions were very clear requesting them not to read aloud, and despite the experimenter begging them not to. Whereas the same-different task, in principle, requires only the orthographic-visual analyzer [50], because they were reading aloud, these children were actually using the sublexical route. Indeed, in doing so, they made many errors in the same-different task (DG: 16%, SY: 20%). These participants were excluded from the analyses and from Fig 7, seeing as they did not perform the task according to instructions, but their performance and the difference between their performance and that of the other participants is informative, pointing to the sublexical route as the source of vowel errors.

#### 3.8.2 Written word comprehension task: Word association

We assessed the comprehension of written words using a word association task. The task included 30 items. Each item was comprised of 4 words: a target word and 3 words from which the participant needed to select one that is semantically related to the target. The target word allowed for at least two different vowel errors that can create existing words. The three options included one word that is semantically related to the target word (e.g., for the target word "sıpa", colt, the semantically related word was "eşek", donkey). The two distractor words were semantically related to possible words resulting from vowel errors in the target word. For example, “sıpa” can be read with vowel migration as “sapı” (grip+acc) and with vowel substitution as “sopa” (stick), so the distractors were “tutacak”(handgrip) and “çubuk” (rod). We balanced the type of errors for the various options (so that sometimes the distractors were related to a word resulting from vowel substitution, sometimes vowel omission, vowel migration, or vowel addition).

The target word was presented in orange on the left, and the three options were presented in black, one above the other to its right, in random order. We requested the participants to circle the word that was most related to the target word, without reading any of the words aloud. (Two participants, SS and BM, were very bored when they got to this task, which was the last task in a long testing session, and the examiner reported that they were marking responses randomly without reading the words, so they were excluded from the analysis for this task).

**Fig 8:**
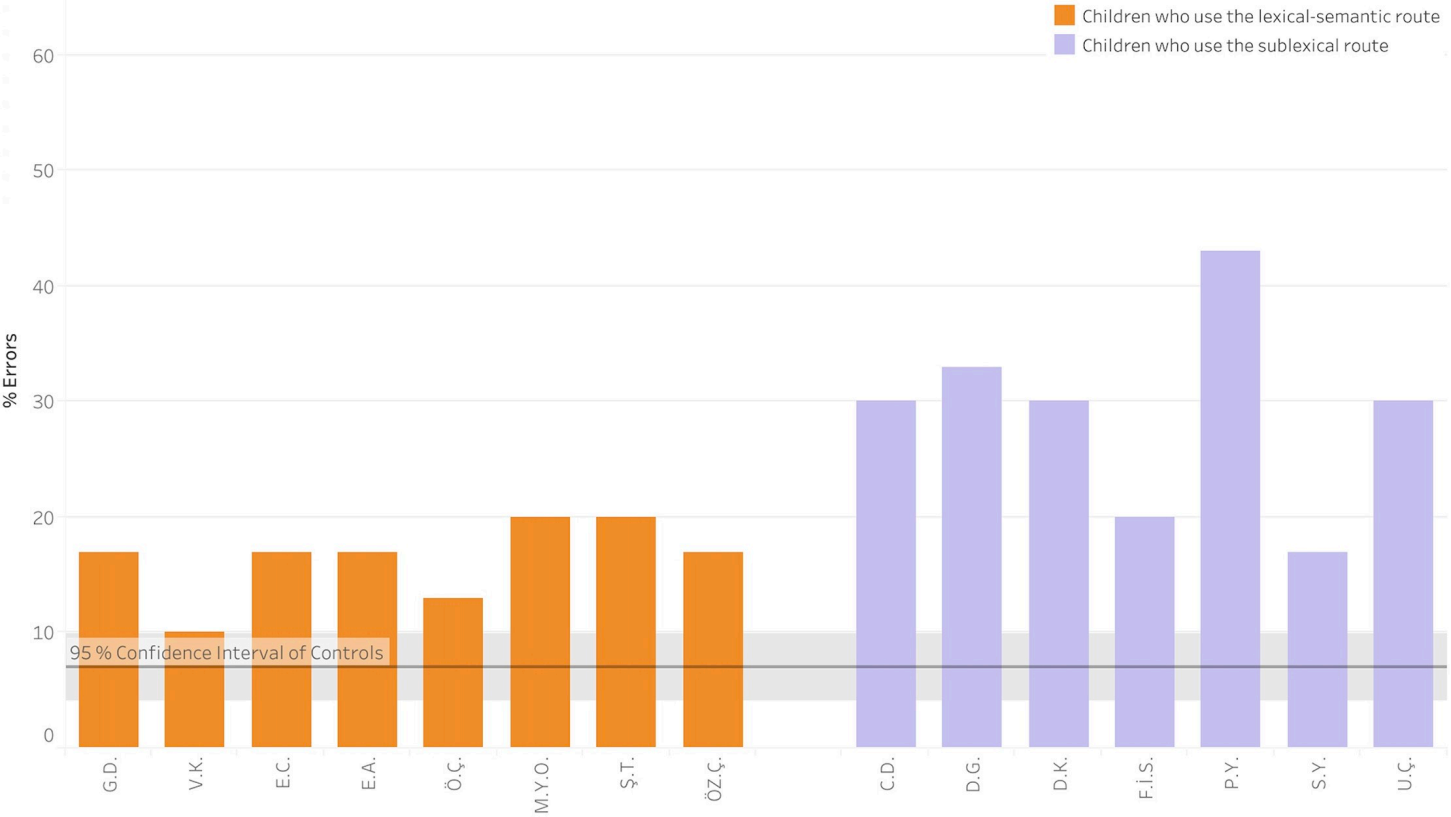
Written word comprehension task with vowel distractors–percentage errors of the participants with vowel dyslexia in comparison to controls mean and 95% CI around it.

In this task, we saw a very clear effect of the route through which the participant is performing the task on the vowel error rate. Children who could use the lexical-semantic route: orthographic-visual analyzer—orthographic input lexicon–semantic system to perform the task performed far better than children who had to perform it via the sublexical route (see Fig 8). Six of the eight children who could use the lexical-semantic route performed similarly to the control group (who made an average of 8.1% errors, *SD* = 5.9%), with a *p <* .05 threshold of 19%.

In contrast, participants who were forced to perform the comprehension task via the sublexical route, in which vowel processing was impaired, made significantly more vowel errors than the controls on this task. One source of using the sublexical route is surface dyslexia. CD, FIS, SY, DK, and PY had surface dyslexia (see section 3.4.3), and hence they had to perform the comprehension task via the vowel-impaired sublexical route; DG, SY, and UÇ were reading aloud despite the request to read silently, so they were also using the sublexical route to perform this task. And indeed, all these participants who were using the sublexical route to perform the task (except one of the oral readers, SY) made significantly more errors than the controls on this task (*t*’s > 2.03, *p’s* < .05).

#### 3.8.3. Interim summary: Silent reading

The results of the same-different decision task indicated that when the participants with vowel dyslexia do not need the sublexical route to perform a task, they do not make vowel errors. The same-different task requires only the orthographic-visual analyzer, and therefore they all succeeded in the task and were able to detect differences in vowels between the words. The two children who did use the sublexical route to perform this task made many vowel errors in it. The same could be seen in the performance on the written word comprehension task: children who could use the lexical-semantic route to perform the task did not make more vowel errors than controls, whereas the children who were using the sublexical route for this task made vowel errors in it.

These results point to the sublexical route as the locus of impairment in the reading model giving rise to vowel dyslexia.

### 3.9. Ruling out the phonological output buffer as the locus of vowel dyslexia

To further explore the locus of impairment that gives rise to vowel dyslexia and to examine an alternative explanation according to which vowel errors resulted from a deficit in the phonological output buffer, we assessed these children’s phonological output using a task of repetition of words that they had read with a vowel error. We also analysed their spontaneous speech and examined whether they made vowel errors in their speech. Three of the participants also participated in a nonword repetition task.

#### 3.9.1. Repetition of words they had read with vowel errors

For each of the 17 children with vowel dyslexia, we selected 10 of the words that they had read with a vowel error and asked them to repeat these words.

All of the 17 participants with vowel dyslexia performed the word repetition task flawlessly, with no vowel error, and in fact, with no other error either.

#### 3.9.2. Analysis of spontaneous speech

We also analysed the participants’ spontaneous speech in free conversation with the examiner during the testing sessions. Each of the vowel dyslexia participants produced 11–52 spontaneous sentences (*M* = 24, *SD* = 16) during the first testing session.

This analysis indicated that none of the vowel dyslexia participants made any vowel error in spontaneous speech.

#### 3.9.3. Nonword repetition

Three of the children (SY, SS, PY) were also tested with a standardized nonword task [51] and their performance was within the norm for their age.

These results indicate that the participants had no vowel errors in phonological output tasks that did not involve reading, supporting our conclusion that the deficit lies in the sublexical route for reading aloud and excluding the possibility that their deficit originated from a deficit in the phonological output buffer.

## 4. Discussion

### 4.1. Vowel dyslexia exists

Our study found 55 Turkish-readers who showed vowel dyslexia: they made significantly more errors in vowel letters than age-matched controls. The vowel errors they made were substitutions with other vowels, migrations of vowels–i.e., incorrect placement of a vowel within the word, as well as vowel omissions and additions.

This finding is in line with previous reports of vowel-specific dyslexia in Hebrew [20] and Arabic [9]. However, vowels in Hebrew and Arabic are not consistently represented orthographically (in Hebrew /a/ and /e/ in the middle of the word are usually not represented orthographically; in Arabic short vowels in the middle of the word are usually not represented). Therefore, finding vowel dyslexia also in Turkish, which has a regular orthography that consistently represents vowels, indicates that it is not a result of the special properties of vowel representation in Semitic orthographies, neither is it a result of the ambiguity of vowel letters. As such vowel dyslexia in Turkish supports the idea that vowel and consonant letters are processed separately.

### 4.2. The underlying deficit is in the sublexical route

#### 4.2.1. Evidence supporting a deficit in the sublexical route as the source of vowel dyslexia

A major task of our study was to identify the locus in the word reading process that is impaired giving rise to the patterns of vowel dyslexia of our participants. All tasks and analyses we conducted to discover the locus pointed to one source in the reading process: the sublexical route. Here we summarize all the findings that lead us to this conclusion.

*More errors in nonwords than words*. The participants made significantly more vowel errors when they read nonwords than when they read words (both the initial group of 55 children with vowel dyslexia, and the subgroup of 17 children for whom we conducted the in-depth testing). Because nonwords are read solely via the sublexical route, whereas words are read (also) via the lexical route, this suggests that the sublexical route is the locus of our participants’ vowel dyslexia.

*Relation with surface dyslexia*. Most children with vowel dyslexia in the current study indeed made significantly more vowel errors when they read nonwords than when they read words. However, there were a few children who made vowel errors both in words and in nonwords, with no significant difference between the two. These children were children who were reading both words and nonwords via the sublexical route–because of a deficit in the lexical route–surface dyslexia. The fact that a deficit in the lexical route, forcing the reader to read words via the sublexical route, causes errors in existing words as well, further supports the conclusion that vowel dyslexia errors emerge when the reader is reading via the sublexical route.

This was also evident in the high correlation between the probability of making a vowel error in reading an existing word, and the multiplication of the probability of reading a word via the sublexical route times the probability of making a vowel error when reading via the sublexical route.

*No vowel errors when reading does not use the sublexical route*. A somewhat similar argument comes from the performance of the vowel dyslexia participants in the comprehension task. If, as we claim, the deficit is in the sublexical route, it should manifest itself only when reading aloud via the sublexical route. Vowel errors are not expected in a task that can be performed without reading aloud via the sublexical route. The task of comprehension of written words was one such task. And indeed, children who could perform this task solely via the orthographic-visual analyzer-orthographic input lexicon-conceptual/semantic system made no vowel errors in this task. However, participants who performed this task via the sublexical route, either because they had surface dyslexia that affected their orthographic input lexicon or because they performed this task while reading the words aloud, made vowel errors in this task as well. This forms another corroboration for the tight relation between vowel errors and the sublexical route.

*Nonlexical error responses*. Another kind of support for the sublexical route being the source of vowel dyslexia in our participants comes from their pattern of error responses. A third of their vowel error responses were nonwords.

All the above considerations point to **a deficit in the sublexical route** as the source of our participants’ vowel dyslexia.

#### 4.2.2. Ruling out deficits in other constituents in the model

The sublexical route starts in input components: the orthographic-visual analyzer and the orthographic input buffer. A same-different test, which included word and nonword pairs differing only in vowels, indicated that these input components are not impaired. The participants with vowel dyslexia (at least those who did not do this task reading aloud) performed well in this task and were able to identify the differences between words and nonwords differing only in vowel letters.

The same conclusion was drawn from the written word comprehension task in which the participant needed to identify and position vowels in words correctly, without reading them aloud. The finding that the participants (those who could perform this task without resorting to reading aloud at least) performed well on this task further indicates that their orthographic-visual analyzer and orthographic input buffer are not impaired and are not the source of their vowel dyslexia. Their good performance in the comprehension task also points to intact orthographic input lexicon (for those participants who did not have surface dyslexia) and good access from the orthographic input lexicon to the semantic system. That the vowel dyslexia of our participants did not stem from a deficit in the orthographic input lexicon could also be concluded from the fact that they made vowel errors not only on existing words but also in nonwords (in fact, even more errors in nonwords than in words).

The locus of the deficit causing vowel errors was not the phonological output buffer either. This was seen in the finding that all participants who made vowel errors in reading made no errors when repeating the same words. They also made no vowel errors in spontaneous speech, nor did they make vowel errors in repeating nonwords.

These findings show that the deficit is not in the production part of reading aloud, neither is it in the orthographic-visual analysis input stage. It is rather located in a stage in between, which, as we have demonstrated in Section 4.2.1, is the sublexical route.

### 4.3. Where exactly is the deficit in the sublexical route? A deficit in a vowel buffer in the sublexical route

**Fig 9:**
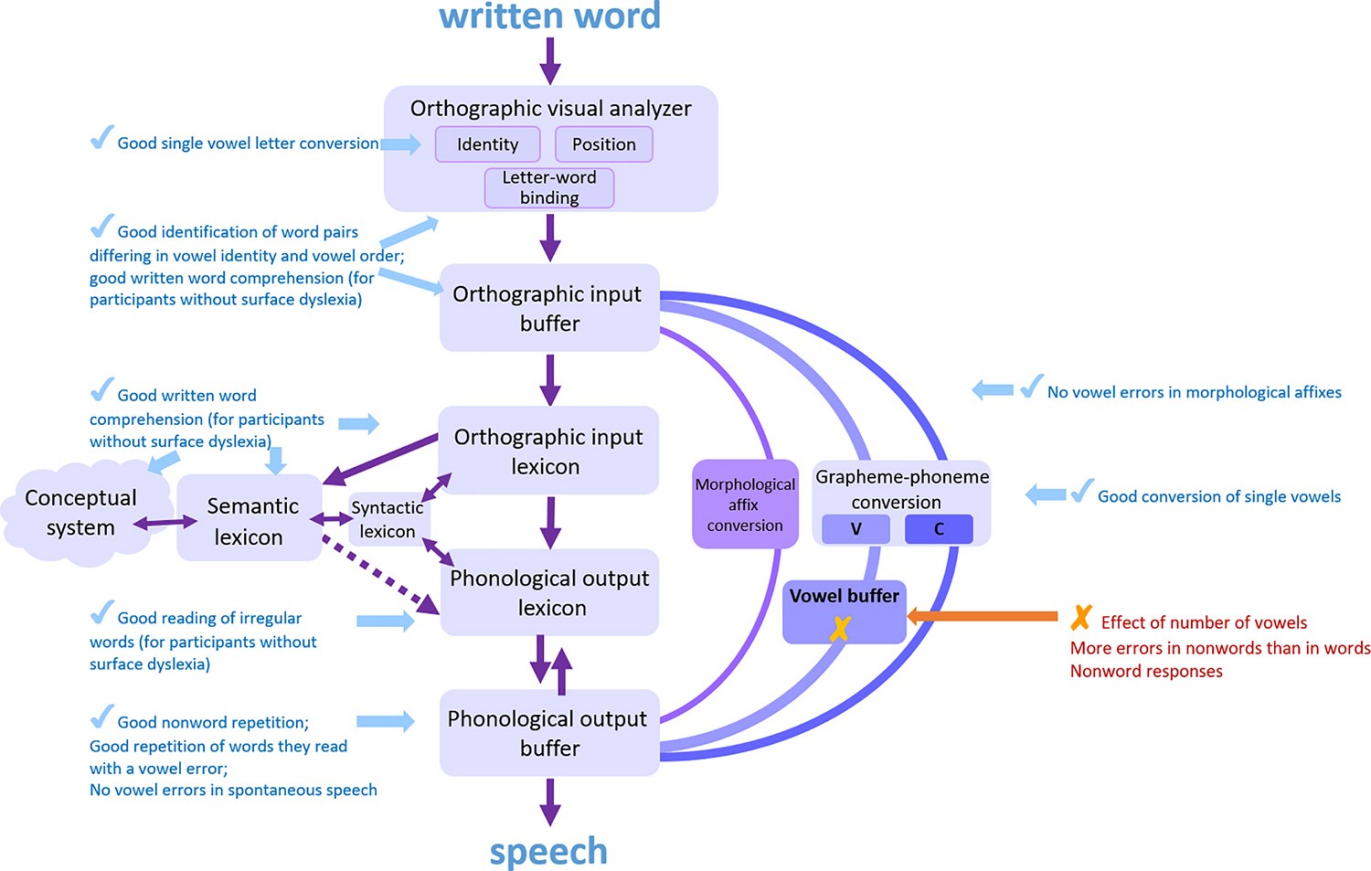
The locus of impairment of our participants in the word reading model, and the evidence regarding the status of each component from the various findings in this study: Blue text and arrows signify tasks in which the participants performed well; orange signifies impaired performance. (Good written word comprehension and good irregular word reading–for vowel dyslexics without surface dyslexia).

The sublexical route uses grapheme-to-phoneme conversion rules to convert letters to phonemes. Is the deficit in vowel dyslexia in the conversion itself? Khentov-Kraus and Friedmann [20] concluded that the vowel dyslexia of their Hebrew-speaking participants was not in the conversion itself but rather in a vowel buffer in the sublexical route that holds the vowel phonemes following their conversion from vowel letters. The pattern of vowel errors of our Turkish-speaking vowel dyslexics points in the same direction (see Fig 9).

*Single letter conversion is fine*. A very straightforward finding ruling out conversion itself as the source of vowel dyslexia comes from a single-letter conversion task. When our participants with vowel dyslexia had to convert single vowel letters to their corresponding vowel phonemes, they did so flawlessly (this task was administered to 13 of the participants).

*Vowel migrations and additions*. Had the deficit in the sublexical route been a deficit in conversion itself, we would have expected vowel substitutions, and maybe also vowel omissions (when the conversion fails, omissions may be an option). However, our participants also made vowel migrations and additions, which cannot be simply accounted for by a deficit in the conversion of vowel letters to vowel phonemes. If, however, during reading via the sublexical route several vowels of the word are held in an impaired vowel buffer, this can account for migrations and additions.

*An effect of the number of vowels*. The error pattern shows buffer properties supporting a deficit in a vowel buffer in the sublexical route. The very clear pattern according to which when the target word included more vowels the participants made more vowel errors (far beyond the per-vowel error probability) is consistent with a deficit in a buffer, which is limited in the number of vowels it can hold and process simultaneously.

This impaired vowel buffer is a vowel-specific buffer that is part of the sublexical route and not the general phonological output buffer. This is indicated by the findings according to which none of the participants had any vowel errors in repeating the words that they had read with a vowel error, nor did they produce vowel errors in nonword repetition or in spontaneous speech.

*Same- vs*. *different-vowels in the buffer*. Another property of our participants’ vowel errors supports the view that the difficulty emerges from a stage in which several vowels are held together. The participants made more vowel errors in words with different vowels than in words in which all the vowels were the same. They made more vowel substitutions in words with different vowels than in words with the same vowels, with about half of the substitutions in words with different vowels being with another vowel that existed in the word. Similarly, when all vowels were the same there were fewer vowel omissions than in words in which there were different vowels. These two findings can be explained if when a buffer holds the same vowel several times it strengthens the activation of this vowel.

Vowel additions seem to corroborate this conclusion from the other direction: there were more vowel additions in words with the same vowel, suggesting, possibly, that a buffer that holds several instances of a vowel may fail to retain the number of instances of this vowel (for similar discussions about double letters in the orthographic output buffer, see Caramazza & Miceli [52]; Tainturier & Rapp [53]; McCloskey et al. [54]). See Haluts et al. [55] for discussion of a neurologically plausible Potts network model of the phonological buffer and the way it accounts for various error types.

All the above considerations indicate that the deficit in the sublexical route is in a vowel-specific buffer, rather than in vowel conversion.

### **4.4. What guides vowel errors when the vowel buffer fails**?

The results also suggested some insights as to what guides the resulting vowel error when the vowel buffer fails. They indicate that when the vowel information is underspecified or partial, vowel frequency and vowel harmony affect the resulting response.

#### 4.4.1. Vowel harmony

The property of vowel harmony in Turkish affected the vowel errors: when a vowel error occurred in reading a (harmonic) word or a nonword, it created a harmonic word 85% of the time. When the target word was non-harmonic, the participants "harmonized" it in 91% of their vowel errors. Namely, when a vowel was added to the word, in most cases it matched the harmony class of the other vowels in the word; when a vowel was substituted, it was substituted with a vowel that was harmonic with the other vowels of the word. Raman and Weekes [23] reported a similar finding for an acquired dysgraphic patient who, despite his phonological output deficit, rarely violated vowel harmony in suffixes when he wrote morphologically complex words in Turkish.

#### 4.4.2. Vowel frequency

Another factor that guided the types of vowel errors that occurred was vowel frequency. Nost of the substituting vowels (83%) were the three most frequent vowels in Turkish: */a/*, and to a lesser degree */i/* and /*e*/. Similarly, 75% of the vowel additions were of the two most frequent vowels. It may be that in case of uncertainty regarding a vowel, the system uses the most frequent vowels instead. The finding that, once a vowel uncertainty occurred, vowel frequency affected the response but not word frequency, further supports the idea that the deficit is in the sublexical, rather than the lexical, route.

### 4.5. Theoretical implications for the reading model

The results of this study bear theoretical implications for the word reading process. In a recent study, Güven and Friedmann [24] studied letter position dyslexia in Turkish and found that individuals with letter position dyslexia make significantly more transpositions of consonants with other consonants than of consonants with vowels, and of vowels with vowels. Given that letter position dyslexia is a deficit in letter position encoding, in the early stage of orthographic-visual analysis, this finding suggests that the consonant-vowel status of a letter is already available at the early stage of orthographic-visual analysis. The current study provided additional strong evidence for the separate treatment of vowels and consonants also in a later stage of the reading process. The selective impairment of our participants, who made errors in vowel letters but not in consonants, suggests that **vowels and consonants are processed separately**. Given that, as we proved above, their deficit is in the sublexical route, this indicates that the sublexical route treats vowels and consonants separately (in line with the conclusion of the study on vowel dyslexia in Hebrew) [20].

The current findings, according to which the sublexical route processes vowels and consonants separately, also explain the previous findings regarding the separate encoding of vowel and consonant letters already in the orthographic-visual analyzer. If the sublexical route makes this distinction and processes vowels and consonants separately, it makes sense that the orthographic-visual analyzer will provide it with input that is already separate for vowels and consonants, and this is why we find vowel-consonant distinctions already in the orthographic-visual analyzer, which is a purely orthographic stage.

The findings according to which there was an effect of the number of vowels in the word, alongside the good conversion of single vowels and the existence of vowel migrations shed further light on the processing of vowels in the sublexical route. They suggest a stage of vowel-specific buffer in the sublexical route. The finding of the effect of the number of vowel letters on error rate, the existence of migrations, and the tendency to make errors that result from the effect of other vowels in the word indicate that this buffer holds several vowels together during conversion, a view that is different from the letter-by-letter conversion view.

A final important theoretical implication of the results pertains to reading morphologically complex words. Turkish is very rich morphologically, and morphological suffixes may include several letters, including vowels. Our results showed that whereas the participants made many vowel errors in the stems of the words, they rarely made vowel errors in morphological suffixes. Such a result is consistent with Friedmann and Coltheart’s [15] morphological model, according to which the sublexical route includes an affix conversion route, which is separate from the vowel and consonant conversion routes of the stem (see Fig 9). In this morphological route, affixes are treated as wholes, and an orthographic whole affix is converted to a whole phonological affix. If the deficit is specific to the vowel conversion process in the sublexical route, it is clear why vowels inside multi-letter affixes are not affected–because they are treated in a separate sub-route.

### 4.6. Implications for developmental dyslexia in Turkish and other transparent orthographies

#### 4.6.1. Types of developmental dyslexia exist also in Turkish

The first conclusion from this study is that specific types of developmental dyslexia in Turkish exist. Here we reported on developmental **vowel letter dyslexia**, which can be readily identified by vowel letter errors in reading aloud, and we have seen that it is not rare: out of 155 Turkish-speaking dyslexic children whom we tested so far, 55 seemed to have vowel letter dyslexia (with or without additional types of dyslexia). We were also able to identify **surface dyslexia** in the reading of five of our vowel dyslexia participants. These two developmental dyslexia types join a recent paper [24] that reported on another type of developmental dyslexia in Turkish: developmental **letter position dyslexia**, which selectively impairs the encoding of letter position within words.

These three types of developmental dyslexia in Turkish join a growing body of evidence from various languages showing that developmental dyslexia, just like acquired dyslexia, has different types that result from selective deficits in different components of the single word reading model [3, 4, 6, 8–11] (Friedmann & Coltheart [15], for types of developmental dyslexia in Hebrew; and Traficante et al. [56] for types of developmental dyslexia in Italian).

#### 4.6.2. Dyslexia may exist even in transparent orthographies

Finding such dyslexias in Turkish is especially informative, against the background of dyslexia researchers claiming that there are no dyslexias in transparent orthographies (Ardila [35], who claimed that "In phonological (transparent) reading systems (like Spanish) reading problems seem to be absent” p. 444) or, relatedly, that dyslexia in transparent languages can only be detected using reading speed measures ("fluency"), and not by reading errors [32, 34]. Such claims have been made for a variety of transparent orthographies, including German [38], Italian [36], and Turkish [37].

What stands behind these two claims is the fact that in transparent orthographies reading via the sublexical route usually results in correct (and possibly slower) reading (see [57] for a discussion of orthographic depth). We believe that this logic may be appropriate when discussing surface dyslexia in these languages: indeed, readers with surface dyslexia, who cannot read aloud via the lexical route, read via the sublexical route. When they read a word in a transparent orthography via the sublexical route, they may nevertheless read it correctly. Therefore, surface dyslexia may be "clinically silent" in transparent orthographies (a beautiful term coined by Zoccolotti et al. [58]). The problem, however, is that the claim that there are no dyslexias or that there are no reading errors in transparent orthographies has been mistakenly applied to all sorts of dyslexia. However, this logic applies only to surface dyslexia; All other types of dyslexia besides surface dyslexia are not sensitive to the regularity of grapheme-to-phoneme conversion rules in a language, and impairments in orthographic-visual analysis, or selective impairments in the sublexical route, for example, should affect transparent orthographies just like they affect deeper ones, creating dyslexias and inducing reading errors. And indeed, we have identified in this study two types of (developmental) dyslexia, which join the previous type of dyslexia (and another type of acquired dyslexia) that have already been reported for Turkish [31].

#### 4.6.3. Not only fluency: Dyslexia can be detected by error analysis when using the relevant stimuli

As we noted above, researchers of reading in Turkish claimed that Turkish-speaking children with dyslexia do not make more reading errors than their age-matched peers, they only read more slowly [33] or that reading errors are not a good measure of dyslexia in Turkish [36, 37]. The same has been claimed for other transparent orthographies as well [36, 38].

Our results are in sharp contradiction with such claims, as our participants with vowel dyslexia did make reading errors–of a specific sort–all the children with vowel dyslexia made vowel errors: substitutions, migrations, omissions, and additions of vowel letters, especially when they were presented with stimuli sensitive to these errors: nonwords with several vowels. Additionally, once the appropriate stimuli have been presented, in this case, irregular words, there were five children with surface dyslexia who also made phonologically plausible errors in the reading of irregular words, i.e., surface dyslexia errors. Similarly, the participants with developmental letter position dyslexia in Güven and Friedmann [24] made letter position errors, especially when they were presented with migratable words.

These findings indicate that once the relevant types of stimuli are presented to individuals with dyslexia, their dyslexia can be identified not only by their slow reading but according to the errors that they make in reading. The analysis of the error types can then inform us about the exact type of dyslexia the reader has.

#### 4.6.4. Even surface dyslexia can be detected in languages with transparent orthography

As we discussed above, the plausible source of the belief that dyslexia in transparent languages cannot be detected using error measures is in the fact that reading via the sublexical route usually results in correct reading. Indeed, Turkish is a very transparent orthography, with the pronunciation of most words corresponding to the result of their conversion via the sublexical route. Still, even Turkish is not completely transparent. We were able to identify irregular words in Turkish, and when we used these in our further investigations of the properties of vowel dyslexia, we were also able to identify surface dyslexia in 5 of our participants: this is interesting as Turkish is known to be especially transparent, but still surface dyslexia is identifiable in it, on the basis of regularization errors, once the relevant words are presented. Surface dyslexia was identified in other transparent orthographies as well. Some researchers used problems rejecting pseudohomophones in lexical decision tasks [58–61]; Slow reading [58, 59, 62, 63]; A deficit in homophone comprehension [60, 62, 64] and eye movement patterns [60, 62] to detect it in Italian, another very transparent orthography. Several studies [58, 62, 65–68] took it one step further, and after analyzing the generalizations about the position of major stress in Italian, were able to identify irregular words in which the stress violates these generalizations. And indeed, these irregular words allowed them to identify surface dyslexia in Italian using reading errors–errors of stress regularization. Such stress regularization errors were also used to detect surface dyslexia according to errors in Filipino, an additional transparent language [69]. (Tomasino [68] also used words with double letters to identify surface dyslexia in Italian through errors in a lexical decision task).

#### 4.6.5. Even in transparent orthographies readers use a lexical and a sublexical route

Several findings indicate that even though Turkish has a transparent orthography, and for most words reading via the sublexical route yields the correct reading, still reading in Turkish is performed in two routes–a lexical route, through which words that are known to the reader and are stored in the orthographic input lexicon and phonological output lexicon are read, and a sublexical route, in which reading is performed using grapheme-to-phoneme conversion rules. This stands in contrast to some claims, which Katz and Frost [70] termed “the strong orthographic depth hypothesis version”, according to which in orthographically-shallow (transparent) languages readers do not need to use the lexical route and can use the sublexical route primarily or exclusively (see [58, 70] for review).

The first support for the dual-route reading comes from the reading of irregular loan words in Turkish: had there been only one, sublexical route, through which Turkish readers read, we would expect all of them to read irregular words incorrectly. The fact that only 5 of the participants read these words incorrectly (i.e., they had surface dyslexia) and the others were able to read the words correctly indicates that there is a lexical route that allows for the correct reading of irregular words.

Secondly, we found very clear differences between the reading of words and nonwords for all vowel dyslexics who did not have surface dyslexia. Had there been only one reading route, through which both words and nonwords are read, such a difference would not be expected.

Moreover, the finding that the participants with vowel dyslexia (except for those who had surface dyslexia or who read aloud in this task) read correctly and without vowel errors in the comprehension task, in which they were not reading aloud, indicates that they do have a lexical and a sublexical route for reading–when they used only the lexical route–when they did not need to read aloud–they did not make vowel errors. When they were using the sublexical route–for example when they read nonwords aloud- this is when they were making vowel errors.

These results are in line with studies in Italian showing lexical effects in typical readers [71] and in dyslexic readers [72], which also indicate that even in transparent languages reading of known words proceeds via the lexical route. See also [65] for a discussion.

#### 4.6.6. Dyslexia is not necessarily a phonological deficit

Many researchers claim that dyslexia, not only in Turkish, is a phonological deficit [73–75]. Many types of dyslexia have been reported in which the impaired components in the reading process are not related in any way to phonology (e.g., impairments in the orthographic-visual analyzer stage, see Castles and Friedmann [76], for a review). Furthermore, many studies have also reported individuals with developmental dyslexia who do not show any difficulty in processing phonemes, repeating nonwords and morphologically complex words, or in manipulating phonemes [10, 20, 54, 58, 77–80].

Our results join previous studies in refuting the phonological impairment claim and further demonstrate that not all individuals with dyslexia have a phonological deficit. None of the 17 children with vowel dyslexia whom we tested with several additional tests showed a deficit in phoneme production: they did not make phonological errors in repeating the same words they failed to read, neither did they make phonological errors in nonword repetition or in spontaneous speech.

A further indication of preserved phonological abilities pertains to a pivotal phonological property of Turkish–vowel harmony. We have seen that the participants retain this phonological knowledge and it in fact guides their incorrect responses.

Thus, even when the dyslexia results from a deficit in the sublexical route, often called "the phonological route", it may still occur while retaining phonological abilities.

#### 4.6.7. Dyslexia does not necessarily involve a morphological deficit

Along similar lines, we can also reject a general claim according to which individuals with dyslexia have a morphological deficit, made by researchers who studied groups of individuals with dyslexia without analysing their exact types of dyslexia. For example, Shankweiler et al. [81], Joanisse et al. [82] claim that children with dyslexia have a specific deficit in morphology and in the phonological quality of morphemes, causing them difficulties in accessing and manipulating morphological affixes. Egan and Pring [83] stated that individuals with dyslexia have a less-developed orthographic knowledge that affects their ability to convert morphological information in print.

Whereas this may hold for some individuals with dyslexia, it did not apply to the participants in our study. Our results showed that the participants with vowel dyslexia were able to decompose and convert the morphologically complex words correctly and did not make vowel errors in suffixes. These results are in line with the findings of Leikin and Even Zur [84] who also found that individuals with dyslexia were sensitive to the morphonological structure of written words in Hebrew.

### 4.7. Clinical implications

#### 4.7.1. Implications for dyslexia assessment: Sensitive stimuli and error analysis

All the above discussion is relevant for the assessment of dyslexia, in Turkish, as well as in other languages. We have seen that types of developmental dyslexia exist, even in transparent orthographies like Turkish, and that these types of dyslexia can be identified on the basis of knowing which types of stimuli are most sensitive to each type of dyslexia, presenting the relevant types of stimuli, and analyzing error types.

Whereas some researchers claimed that only fluency can be used to detect dyslexia in transparent languages, we have shown that this is not true. Once the relevant stimuli are presented, errors occur, once the error types are analysed, the exact type of dyslexia can be identified. If only fluency is assessed, it only gives two shades of reading: slow, and within the normal speed. Types of dyslexia cannot be discerned if only fluency is considered. However, once error types are considered, types of dyslexia can be identified.

The clinical conclusion from the current study, then, relates to the properties of the stimuli that need to be included in a diagnostic reading list: stimuli that are most sensitive to each type of dyslexia. For vowel dyslexia, the best stimuli would be nonwords that include several vowels. For surface dyslexia, these would be irregular words.

A diagnostic list of words and nonwords that will allow for the diagnosis of the various types of dyslexia in each language would therefore need to include the relevant stimuli for each type of dyslexia. The administration of such a test should then be followed by a detailed analysis of the error types the individual with dyslexia made, and possibly with additional tasks aimed at differential diagnosis of the source of these errors. This will allow the identification of dyslexia type on which a targeted dyslexia treatment can be based.

#### 4.7.2. Implications for treatment

Given that the deficit that underlies vowel dyslexia is in the sublexical route, word reading is affected only when words are read via the sublexical route. Therefore, one immediate implication for treatment is to enhance the ability of individuals with vowel dyslexia to use the lexical route. This can be achieved by filling their orthographic input lexicon and phonological output lexicon with words, by way of intensive exposure to reading as well as by lexical intervention. Once they are able to read a word via the lexical route, their vowel dyslexia will no longer affect their reading of this word.

Another direction, which is more suitable for the more transparent orthographies, is teaching individuals with vowel dyslexia to break longer words (words with several vowel letters) into smaller parts. That way, the task of the vowel buffer becomes easier, and errors are expected to diminish.

Another direction that may be useful is raising the awareness of the person with vowel dyslexia to vowel dyslexia, and to vowels within words, encouraging them to focus on the identity and position of vowel letters in the word they are reading. Future research will hopefully identify the most efficient ways to help people with vowel dyslexia to do so.

## Supporting information

S1 Fig Percentage vowel errors that the 55 participants made in the ÜZÜM word and nonword reading aloud tests. (DOCX)

S1 Table Tests from the *FRİGÜ test battery* used in the current study, the number of items (and length in letters) in each test. (DOCX)

S2 Table Mean percentage vowel and consonant errors that the 55 participants made in the ÜZÜM word and nonword reading aloud tests. (DOCX)

S3 Table Examples for Vowel Errors of the Participants with Vowel Dyslexia in Words and nonwords. (DOCX)
