# Supplementary material for: Vowel dyslexia in Turkish: A window to the complex structure of the sublexical route
Source: PLoS One. 2021 Mar 24;16(3):e0249016. doi: 10.1371/journal.pone.0249016 (PMC7990308; doi:10.1371/journal.pone.0249016)
Supplement: S1 Fig — (DOCX) [file pone.0249016.s001.docx]

**
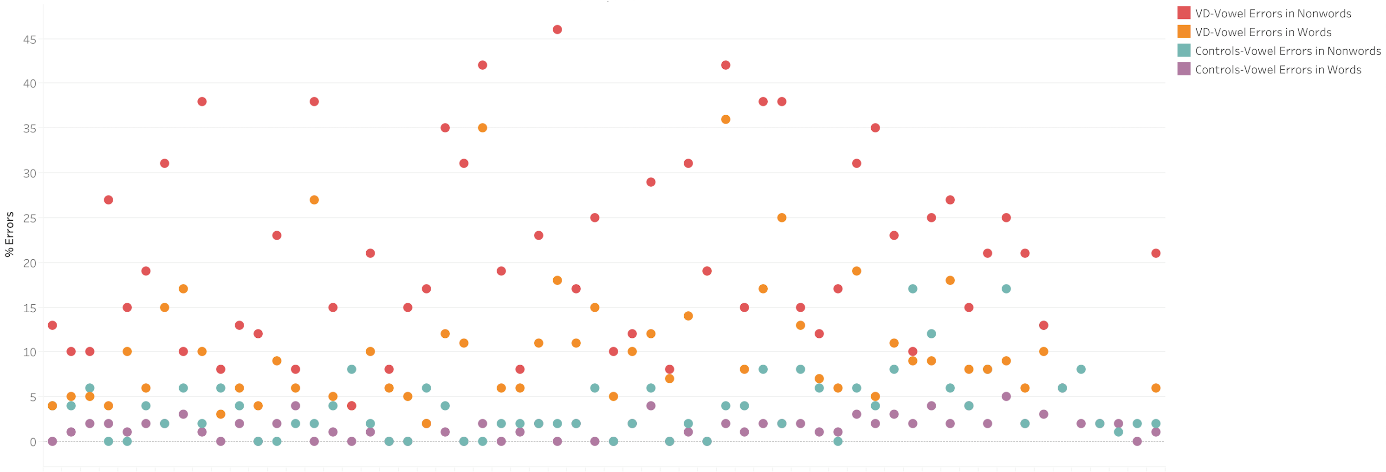
**

**S1 Fig.** Percentage vowel errors that the 55 participants made in the ÜZÜM word and nonword reading aloud tests. The 205 control participants (those who did not complain about reading errors, had no diagnosis of learning disability and were not outliers in the screening test) made an average of 2 (2%) vowel errors (omission, addition, substitution, and migration of vowel letters) in the word reading part of the screening test, and 4 (4%) vowel errors in the nonword reading part.
