# Supplementary material for: Vowel dyslexia in Turkish: A window to the complex structure of the sublexical route
Source: PLoS One. 2021 Mar 24;16(3):e0249016. doi: 10.1371/journal.pone.0249016 (PMC7990308; doi:10.1371/journal.pone.0249016)
Supplement: S1 Table — (DOCX) [file pone.0249016.s002.docx]

**S1 Table.** Tests from the FRIGÜ *test battery* used in the study, the number of items, number of letters, and number of control participants of each test

| Tests | Number of items | Number of letters range, M (SD) | N control participants |
| --- | --- | --- | --- |
| *FRİGÜ* Screening Words  124 migratable  118 for neglect  100 vowel  30 abstract  16 function words  10 irregular | 151 | 2-8  *M* 5.12 (*SD* 1.29) | 205 |
| *FRİGÜ* Screening Nonwords | 42 | 2-9  *M* 5.16, (*SD* 1.62) | 205 |
| *FRİGÜ* Screening Word pairs | 60 | 4-5  *M* 4.88, (*SD* 0.92) | 205 |
| ÜZÜM Words  (Vowel dyslexia tests) | 124 | 4-7 | 60 |
| NANE Irregular Words  (Surface dyslexia tests) | 51 | 4-8 | 60 |
| ÜZÜM Nonwords  (Vowel dyslexia tests) | 52 | 4-6 | 60 |
| ***ÜZÜM vowel dyslexia* *silent reading tasks*** | | |  |
| ÜZÜM Lexical Decision | 82 | 4-6 | 60 |
| ÜZÜM Same-Different | 101 pairs | 4-7 | 60 |
| ÜZÜM Written Comprehension | 30 |  | 60 |
