# Supplementary material for: Vowel dyslexia in Turkish: A window to the complex structure of the sublexical route
Source: PLoS One. 2021 Mar 24;16(3):e0249016. doi: 10.1371/journal.pone.0249016 (PMC7990308; doi:10.1371/journal.pone.0249016)
Supplement: S2 Table — (DOCX) [file pone.0249016.s003.docx]

**S2 Table.** Mean percentage vowel and consonant errors that the 55 participants made in the ÜZÜM word and nonword reading aloud tests

|  | Vowel Errors in Words (SD) | Consonant Errors in Words (SD) | Vowel Errors in Nonwords (SD) | Consonant Errors in Nonwords (SD) |
| --- | --- | --- | --- | --- |
| Vowel Dyslexia Group (N=55) | 11  (7) | 5  (3) | 21  (11) | 4  (5) |
| Control Group (N=60) | 2  (1) | 2  (2) | 4  (4) | 2  (4) |
