# Supplementary material for: Vowel dyslexia in Turkish: A window to the complex structure of the sublexical route
Source: PLoS One. 2021 Mar 24;16(3):e0249016. doi: 10.1371/journal.pone.0249016 (PMC7990308; doi:10.1371/journal.pone.0249016)
Supplement: S3 Table — (DOCX) [file pone.0249016.s004.docx]

**S3 Table. Examples for vowel errors that the participants with vowel dyslexia made in target words and nonwords**

| Subsitution | Omission | Migration | Addition | Subsitution: Harmonic to Nonharmonic | Subsitution: Nonharmonic to Harmonic |
| --- | --- | --- | --- | --- | --- |
| Words |  |  |  |  |  |
| içini-içeni | dizene-dizen | peşin-pişen | pişen-pişeni | delme-delma | aslen-eslen |
| yenile-yenili | içeni-içen | veda-vade | salı-asalı | halay-helay | vida-vide |
| bıkar-bikar | çatılı-çatlı | gerindi-girendi | bale-baale | arkam-erkam | kaset-keset |
| takınık-tıkınık | kasılı-kaslı | bale-bela | ayran-ayaran | akran-ekran | koli-kolu |
| gerindi-girindi | dileme-dilme | tıkalı-tıkıla | siren-esiren | etli-atli | idare-idere |
| perili-pirili | bilime-bilim | pireli-perili | kanlı-kanılı | ense-ensa | silah-sileh |
| elime-elimi | esirime-esrime | sıralı-sırıla | elim-elime | kenetli-kanetli |  |
| sanı-sani | içeni-çeni | bıkar-bakır | benli-benili | sakın-sakin |  |
| Nonwords |  |  |  |  |  |
| girelem-girelim | kartu-kart | salem-selam | ikle-ikele |  |  |
| sırıla-sırala | çarek-çark | kıpa-kapı | amni-amani |  |  |
| askalı-askılı | arıka-arka | saken-sekan | kurat-ukurat |  |  |
| kesa-kese | kerük-kerk | kazo-koza | pilto-pilito |  |  |
| çarek-çarık | ıradan-ırdan | belgö-bölge | amni-amini |  |  |
